# Fluorination-enabled optimal morphology leads to over 11% efficiency for inverted small-molecule organic solar cells

**DOI:** 10.1038/ncomms13740

**Published:** 2016-12-19

**Authors:** Dan Deng, Yajie Zhang, Jianqi Zhang, Zaiyu Wang, Lingyun Zhu, Jin Fang, Benzheng Xia, Zhen Wang, Kun Lu, Wei Ma, Zhixiang Wei

**Affiliations:** 1 CAS Key Laboratory of Nanosystem and Hierarchical Fabrication, CAS Center for Excellence in Nanoscience, National Center for Nanoscience and Technology, Beijing 100190, China; 2 State Key Laboratory for Mechanical Behavior of Materials, Xi'an Jiaotong University, Xi'an 710049, China; 3 University of Chinese Academy of Sciences, Beijing 100049, China

## Abstract

Solution-processable small molecules for organic solar cells have attracted intense attention for their advantages of definite molecular structures compared with their polymer counterparts. However, the device efficiencies based on small molecules are still lower than those of polymers, especially for inverted devices, the highest efficiency of which is <9%. Here we report three novel solution-processable small molecules, which contain π-bridges with gradient-decreased electron density and end acceptors substituted with various fluorine atoms (0F, 1F and 2F, respectively). Fluorination leads to an optimal active layer morphology, including an enhanced domain purity, the formation of hierarchical domain size and a directional vertical phase gradation. The optimal morphology balances charge separation and transfer, and facilitates charge collection. As a consequence, fluorinated molecules exhibit excellent inverted device performance, and an average power conversion efficiency of 11.08% is achieved for a two-fluorine atom substituted molecule.

## Abstract (web summary)

Organic solar cells based on solution-processable small molecules still lag behind their macromolecule counterparts. Here, Deng *et al*. develop molecular donors to pair with PC_71_BM and study how the degree of fluorination impacts the morphology of the heterojunction and the efficiency of the devices.

Organic solar cells (OSCs) have attracted intense attentions due to their potential for solution processing cheap and flexible devices. Comparing with conventional device architectures, inverted devices exhibit improved environmental stability and more preferable for industrial applications^1^. Polymer solar cells with power conversion efficiency (PCE) higher than 10% are mainly obtained through inverted devices^2^,^3^,^4^, and the highest efficiency has reached 11.7% (ref. 5). Solution-processable small molecules for OSCs have attracted intense attentions for their advantages of high purity and definite molecular structures compared with polymers^6^,^7^,^8^,^9^,^10^,^11^. To date, the PCE based on small molecules has reached 10% by conventional devices^8^,^12^. However, attempts to develop inverted devices for small molecules are not as successful as that of polymers^13^,^14^,^15^,^16^, and the highest PCE reported is 8.84% (ref. 17), which is much lagging behind their polymer couterparts.

To obtain highly efficient OSCs, decisive parameters, namely, open circuit voltage (*V*_oc_), fill factor (FF) and short-circuit current (*J*_sc_) should be enhanced. Each parameter could be expressed as following general formula:


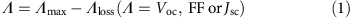


A proved successful strategy in molecular design is to increase 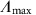. The approaches involve increasing ionization potential of the donor (IP_D_), narrowing bandgap and enhancing mobility to achieve high *V*_oc_, *J*_sc_ and FF, respectively^18^,^19^,^20^. However, IP_D_ enhancement would lead to bandgap increase^19^, and (*V*_oc_ × *J*_sc_)_max_ is limited by Shockley–Queisser model. To further maximize PCE, an alternative strategy is to minimize 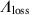. The main loss of *V*_oc_ is related to the disorder arrangement of donors/acceptors and their poor contact with electrode^21^,^22^,^23^; the main losses of FF and *J*_sc_ are ascribed to the recombination induced by undesirable distribution of intermix and crystalline phases, interfacial traps and poor domain purity^24^,^25^. Consequently, all losses are related to the bulk-heterojunction (BHJ) morphology, the optimization of which could simultaneously minimize *Λ*_loss_ for all three parameters.

The adjustment of the donor–acceptor (D:A) interaction is an essential approach to optimize the BHJ morphologies. A difference in surface free energies between donors and acceptors would result in a repulsive interaction (lower miscibility), which acts as the internal driving force to form phase separation^26^,^27^. Hence, a proper disparity of surface free energies is significant to achieving optimized lateral morphology. Furthermore, a lower surface free energy of donors in comparison with acceptors would induce surface enrichments and vertical phase separation^28^, both of which could effectively decrease losses of *V*_oc_, *J*_sc_ and FF through suppression of recombination by modifying interface contact, forming charge-blocking regions and facilitating charge collection^24^,^28^.

In this paper, three medium bandgap molecules are designed and synthesized with thiophene-substituted benzodithiophene (TBDT) as a core, 2-(thiophen-2-yl)thieno [3,2-*b*]thiophene as π-bridges and end-capped with 1H-indene-1,3(2H)-dione, 4-fluoro-1H-indene-1,3(2H)-dione or 4,7-difluoro-1H-indene-1,3(2H)-dione; these molecules are abbreviated as **BTID-0F**, **BTID-1F** and **BTID-2F** (Fig. 1a). With incremental introduction of fluorine to end-capped units, the PCE for inverted devices increases from 8.30% for **BTID-0F** to 10.4% for **BTID-1F** and to 11.3% for **BTID-2F**. A hierarchical morphology with higher domain purity, enhanced surface enrichment and more directional vertical phase distribution is induced by fluorination, thereby *V*_oc_, *J*_sc_ and FF are increased simultaneously.

## Results

### Design and characterization of small molecules

To benefit the charge carrier transport, a two-dimensional donor unit, TBDT was selected as the core^29^. A new π-bridge, namely, 2-(thiophen-2-yl)thieno[3,2-*b*]thiophene, was designed with two advantages: first, it introduces stronger aromaticity units of thieno[3,2-*b*]thiophene to increase IP_D_, aiming to increase attainable *V*_oc_. Second, it presents an inner gradient-decreased electron density distribution (Supplementary Fig. 1), benefited for backbone hole transfer. To lower the miscibility and increase dielectric constant, 1H-indene-1,3(2H)-dione was selected as acceptor because of its stronger polarity and aromatic difference in comparison with [6,6]-phenyl-C_71_-butyric acid methyl ester (PC_71_BM)^26^. To decrease molecular surface free energies and miscibility with PC_71_BM, we introduced fluorine atoms^30^ to the end-capped acceptor units and obtained two new acceptor units, namely, 4-fluoro-1H-indene-1,3(2H)-dione and 4,7-difluoro-1H-indene-1,3(2H)-dione. Various studies have discussed fluorination effects on photovoltaic performance but most focused on modifying molecular internal part to stabilize molecular conformation^31^,^32^. In addition, the positions of alkyl chains were designed to prevent backbone torsion to highest extent. The different alkyl chains attached to TBDT units for **BTID-0F**, **BTID-1F** and **BTID-2F** were used to ensure their sufficient solubility. The synthesis routes of the three molecules are shown in Supplementary Fig. 2. The dielectric constants of **BTID-0F**, **BTID-1F** and **BTID-2F** are calculated as *ca*. 4.0 (Supplementary Table 1, Supplementary Fig. 3a), which was a high value among the conjugated small molecules or polymers^33^.

**Figure 1:**
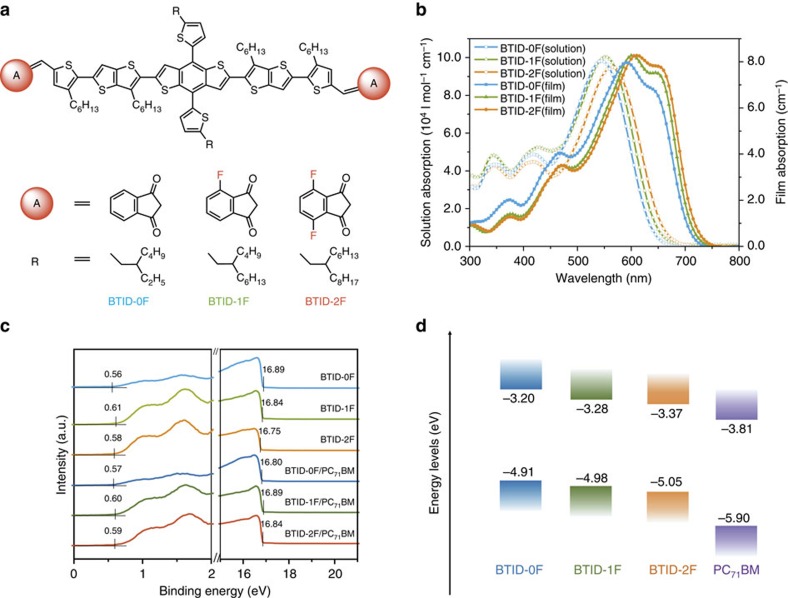
Molecular structures and properties. (**a**) Small molecular structures; (**b**) solution coefficient of samples in chloroform (left *Y* axis) and film (right *Y* axis); (**c**) UPS results of the pristine films and blended films with PC_71_BM; and (**d**) molecular energy levels measured from UPS, the lowest unoccupied molecular orbital (LUMO) levels calculated from optical bandgap and UPS.

Absorption spectra of solution and films are shown in Fig. 1b. The absorption coefficient in solution of the three molecules denoted the competition between the content of alkyl chains and fluorine atoms. Although, four fluorine atoms were introduced into **BTID-2F**, the alkyl chain (no contribution to absorption) content of which was the highest. Thus, **BTID-2F** exhibited the lowest absorption coefficient in solution, whereas **BTID-1F** showed the highest. Furthermore, introduction of fluorine not only enhanced absorption coefficient gradually but also slightly redshifted the absorption spectrum. From solution to films, all the molecules clearly manifested *ca*. 70 nm redshifts, illustrating good aggregation in films. With the increase of fluorine atoms, the intensity ratios of the shoulder peak (attributed to π–π stacking) to the absorption peak from internal charge transfer increased. The different tendencies of film absorption coefficient and the solution absorption coefficient further revealed that fluorination led to dense and ordered π–π stacking.

Ultraviolet photoelectron spectroscopy (UPS) was carried out to obtain IP_D_ in pristine films and blends with PC_71_BM on PEDOT:PSS/ITO substrates (Fig. 1c)^34^, and the IP and EA (electron affinity) of PC_71_BM were obtained from literatures^35^. The IP_D_ of the three pristine films were 4.91, 4.98 and 5.05 eV, respectively, indicating fluorination of the end acceptors increase IP_D_ comparing with those of fluorine atoms attached to the internal part of polymers^36^,^37^. The detailed −IP_D_ and −EA_D_ of the three molecules are shown Fig. 1d, and the EA_D_ was calculated from: 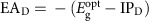 (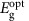 is optical energy bandgap). However, after blending with PC_71_BM, the IP_D_ were changed to 4.98, 4.92 and 4.95 eV for **BTID-0F**, **BTID-1F** and **BTID-2F**, and their corresponding changed values were 0.09, −0.05 and −0.09 eV. The change from pristine to blended films was mainly due to the shifts in work function (Fig. 1c) indicating dipole polarity differs after PC_71_BM addition. Changes in dipole direction after fluorination were probably due to various surface enrichment and vertical distributions, which will be discussed in the later section. Moreover, the molecules based on the new designed π-bridge exhibited low highest occupied molecular orbital (HOMO) levels (measured by cyclic voltammetry, Supplementary Fig. 3b,c), which is the basis for obtaining high *V*_oc_ in BHJ OSCs^5^.

To check the miscibility and molecular surface free energies, the contact angles of chloroform solution of three materials were measured on ZnO/ITO and PEDOT/ITO substrates (Supplementary Fig. 3d,e). The increase of contact angles for the fluorinated molecules indicated fluorination lowered the surface free energies of small molecules. On the other hand, the increase of deviation in contact angles between fluorinated molecules and PC_71_BM indicated fluorination also reduced the miscibility of small molecules with PC_71_BM. These results implied that surface free energies and miscibility were successfully adjusted through molecular design.

### Fabrication and performance of inverted solar cells

**Figure 2:**
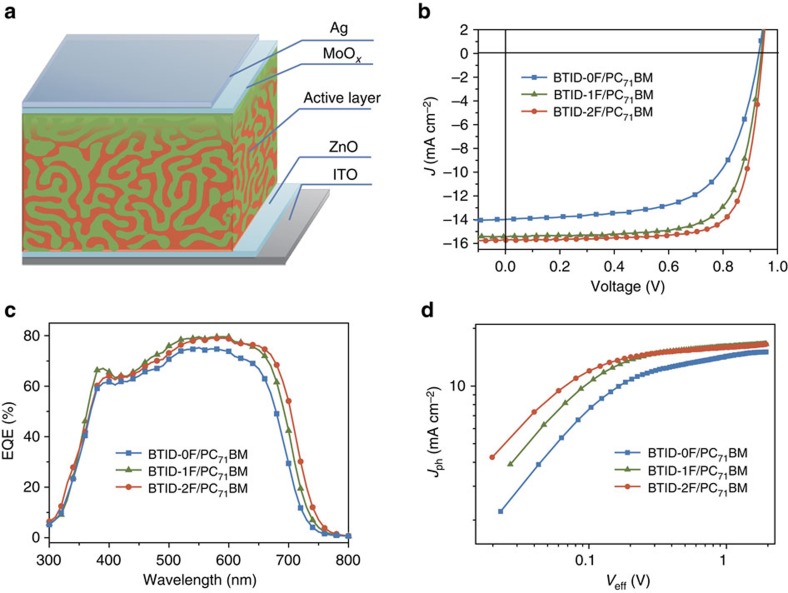
Device structures and photovoltaic properties for inverted solar cells. (**a**) Structures of inverted device; (**b**) optimized *J*–*V* curves for inverted devices; (**c**) EQE corresponding to devices in **b**,**d** photocurrent density versus effective voltage (*J*_ph_–*V*_eff_) characteristics for devices under constant incident light intensity (AM 1.5 G, 100 mW cm^−2^).

To investigate the photovoltaic properties of the three small molecules, devices with a structure of ITO/ZnO/active layer/MoO_*x*_/Ag were fabricated (Fig. 2a). The highest PCE for **BTID-0F**, **BTID-1F** and **BTID-2F** were 8.30, 10.4 and 11.3%, and other detailed parameters are shown in Fig. 2b,c and Table 1, and the optimization process of *D*:*A* ratios is shown in Supplementary Table 2. The device based on **BTID-2F** was certified at an accredited laboratory, certifying a PCE of 11.0% (Supplementary Fig. 4a–e). Notably, the active layers of all devices were obtained without any additives and post-treatment, which were facilitated for future industrial manufacturing^38^.

The high efficiencies of the three materials were ascribed to simultaneous increment in *V*_oc_, *J*_sc_ and FF. The high *V*_oc_ was in good agreement with the IP_D_ of their blends obtained by UPS. As calculated from the formula: 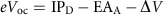 (refs 39, 40), the *V*_oc_ losses (Δ*V*) based on all three small molecules were *ca.* 0.2 eV (−IP_D_ and −EA_A_ values shown in Fig. 1d), which were much less than empirical value 0.3−0.5 eV reported^39^,^40^. The low *V*_oc_ loss were attributed to their high dielectric constant (Supplementary Fig. 3a, Supplementary Table 1)^33^,^40^ and ideal morphologies, which will be discussed in following sections. The *J*_sc_ of **BTID-2F** and **BTID-1F** cells was higher than that of **BTID-0F** cells partly because of the larger photocurrent generated in the red region (Fig. 2c). On the other hand, the enhancements of FF and partial *J*_sc_ for **BTID-2F** and **BTID-1F** compared with **BTID-0F**, illustrated lower loss of FF and *J*_sc_ with fluorination. This phenomenon could be supported by the relation of photocurrent density (*J*_ph_) versus effective voltage (*V*_eff_) (Fig. 2d), where *J*_ph_=*J*_L_*−J*_D_ (*J*_L_ and *J*_D_ are the current density under illumination and in the dark) and *V*_eff_=*V*_0_*−V*_a_, (*V*_0_ is the voltage at *J*_ph_=0 and *V*_a_ is the measured voltage under different current density). The ratios of *J*_ph_*/J*_ph,sat_ are used to judge the overall efficiency of exciton dissociation and charge collection^2^,^41^. Under short-circuit condition, the ratios were 0.93, 0.95 and 0.96 for **BTID-0F**, **BTID-1F** and **BTID-2F**, suggesting the effective exciton dissociation of the three molecules, especially for **BTID-1F** and **BTID-2F**. Under maximal power output circumstances (*V*_eff_=0.2 V), the ratios of *J*_ph_*/J*_ph,sat_ were 0.71, 0.83 and 0.86, respectively, indicating a considerably higher charge collection and lower bimolecular recombination after fluorine substitution. The superior *J*_ph_*–V*_eff_ characteristics clearly demonstrated that fluorination could reduce bimolecular recombination, thereby improving *J*_sc_ and FF simultaneously.

### Hierarchical morphology

**Figure 3:**
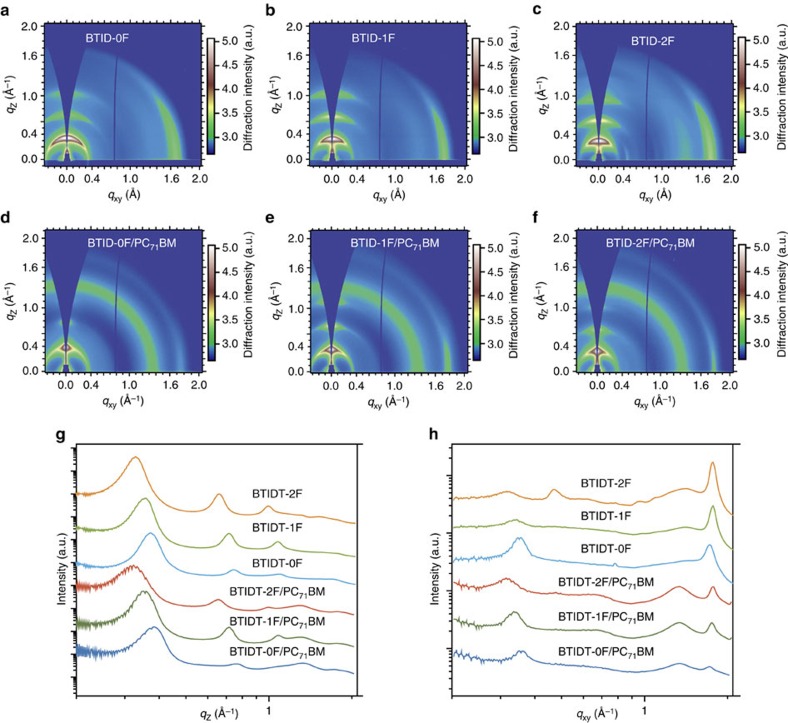
Microstructures of pristine and blend films. (**a**–**f**) GIWAXS images in pristine films on Si substrate and GIWAXS images in blends films on ZnO/Si substrates; (**g**) corresponding out-of-plane curves; and (**h**) corresponding in-plane curves.

The small loss in *J*_sc_, FF and *V*_oc_ for devices based on the three molecules and their variation in loss should be ascribed to the optimized but different lateral/vertical phase distributions. To study morphologies in the lateral direction, we characterized small molecules/PC_71_BM blending films on ZnO/Si substrates and pristine films on Si substrates by grazing incidence wide-angle X-ray scattering (GIWAXS) (Fig. 3a–f), and their corresponding one-dimension curves are shown in Fig. 3g,h. Whether in pristine or blended films, all three molecules exhibited preferable edge-on molecular packing orientation with a small ratio of face-on packing orientation, because evident multiple higher-order (*h*00) reflections in the out-of-plane direction and an evident (010) reflection of π–π stacking in the in-plane direction were observed for all samples. From the calculated face-on to edge-on ratios (Supplementary Table 3), it could be easily found: in pristine films, molecules adopted a more favourable edge-on packing mode; while in blends, the ratios of face-on to edge-on orientation were similar for the three molecules. The differences in *d*-spacing in the (100) direction were ascribed to the varying lengths of the alkyl (corresponds to the short axis periodicity). The π–π stacking distance of **BTID-0F**, **BTID-1F** and **BTID-2F** were 3.63, 3.57 and 3.55 Å, illustrating a more condensed stacking in the π–π direction after fluorination; a result was consistent with the absorption in films. The coherence lengths calculated from the π–π stacking (010) peaks were 51.0, 67.3 and 70.4 Å for **BTID-0F**, **BTID-1F** and **BTID-2F**, suggesting fluorination of end-capped units increases order range. In comparison with pristine film (Fig. 3a–c), additions of PC_71_BM decreased the coherence length by 13.2, 10.4 and 2% for **BTID-0F**, **BTID-1F** and **BTID-2F**, indicating that fluorination decreases the influence of PC_71_BM on molecular aggregation, especially for **BTID-2F**; the phenomenon could be ascribed to their decreased miscibility with PC_71_BM. In addition, PC_71_BM in all blends exhibited strong aggregation with coherence lengths *ca.* 20 Å (Supplementary Table 3). The good aggregations for PC_71_BM and small molecules reduced interfacial energy disorder, which was beneficial for further lowering loss of *V*_oc_ (ref. 22).

**Figure 4:**
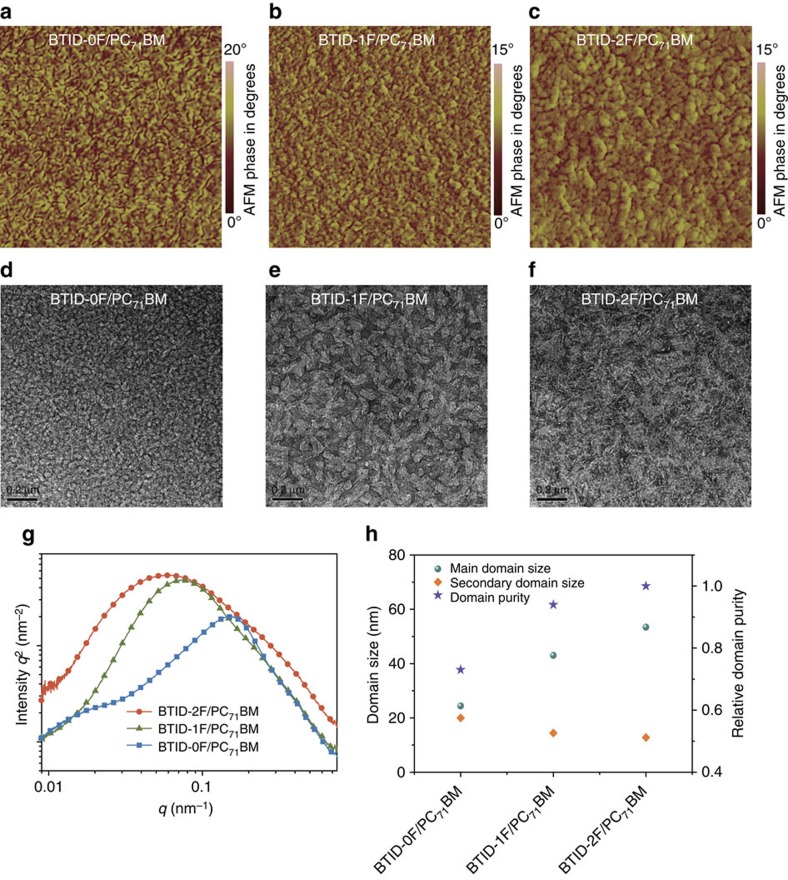
Lateral morphologies and microstructures of blend films on ZnO/ITO substrates. (**a**–**c**) AFM phase images; (**d**–**f**) TEM images; (**g**) RSoXs profiles; and (**h**) relative domain purity and domain sizes.

The morphologies were further investigated by atomic force spectroscopy (AFM) and transmission electron microscopy (TEM). The domain in AFM phase images (Fig. 4a–c) increased in size after fluorinations in accordance with their enhanced crystalline. As seen in TEM images (Fig. 4d–f), nanostructures were observed for **BTID-0F**/PC_71_BM blends with diameters *ca.* 25 nm. After fluorination, the diameters of nanostructure increased evidently for **BTID-1F**/PC_71_BM blends because of increased order range. Interestingly, with a further increase in fluorine atoms, an evident network of whiskers with diameter *ca.* 15 nm was observed, interpenetrating in the larger domains.

Resonant soft X-ray scattering (RSoXS) was employed to investigate the above mentioned fine microstructure (Fig. 4g). With increment of fluorine atoms, the dominated domain size increased from 24 to 43, and finally to 53 nm, as in good agreement with the increased crystallinity. However, by carefully analysing the RSoXS profiles, we could see that the scattering distribution could be fitted by two log-normal functions, with the other peak in the longer *q*-values: *ca.* 0.25 nm^–1^ for **BTID-2F** (corresponding to domain size of 12.8 nm, calculated from plot fitting in Supplementary Fig. 5) and a less evident interference appeared at *ca.* 0.22 nm^–1^ for **BTID-1F** (corresponding to domain size of 14.4 nm). As for **BTID-0F**, no obvious difference was found between the two fitting domain sizes (24 and 20 nm). In other words, **BTID-2F**/PC_71_BM and **BTID-1F**/PC_71_BM blends demonstrated formation of a hierarchical morphology with secondary domain sizes, and the secondary domain size was closed to the exciton diffusion length of *ca*. 10 nm (Fig. 4h). Moreover, the relative domain purity for **BTID-0F**, **BTID-1F** and **BTID-2F** was calculated as 0.70, 0.93 and 1, in good agreement with increased crystallinity and decreased miscibility by increased fluorine introduction (Fig. 4g,h). The hierarchical morphology is reported to well balance domain size and purity to facilitate charge separation and transfer; the smaller donor phase accounts for charge separation, whereas the larger donor phase is responsible for charge transport^42^,^43^. Therefore, the hierarchical morphology consisting 10–20 nm structure and enhanced domain purity could increase FF and *J*_sc_ simultaneously.

### Surface enrichment and vertical phase distribution

**Figure 5:**
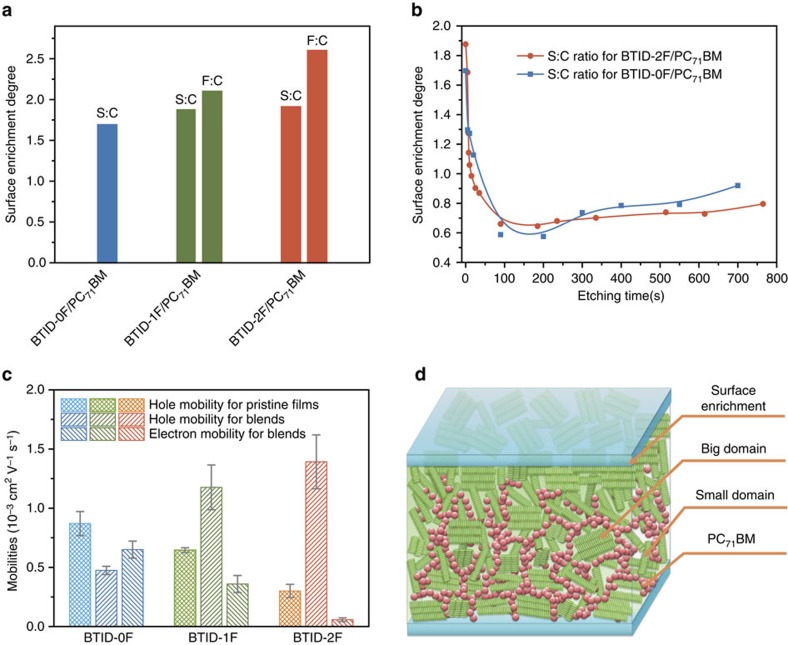
Vertical morphologies of blended films. (**a**) Surface enrichments of BTID-0F/PC_71_BM, BTID-1F/PC_71_BM and BTID-2F/PC_71_BM on ZnO/ITO substrate; (**b**) in-depth XPS profiles of BTID-0F/PC_71_BM and BTID-2F/PC_71_BM on ZnO/ITO substrates, the line were obtained from fitting all the S:C value from various etching time; (**c**) carrier mobilities of blends and pristine films, the average values were obtained from the mobility measured from the thickness between 100 and 130 nm, the error bars come from the mobility value from thickness differences and their measurements errors; and (**d**) schematic illustrations of lateral and vertical phase distribution of BTID-2F/PC_71_BM.

X-ray photoelectron spectroscopy (XPS) was carried out to study surface enrichment and vertical phase distribution. In the blends of small molecules: PC_71_BM, sulfur and fluorine atoms could be used as characteristic elements of the small molecules due to the absence of the two elements of PC_71_BM. A parameter named ‘surface enrichment degree' was introduced to characterize surface enrichment, which was equal to divide the S:C (or F:C) ratio measured through XPS by the ideal S:C (or F:C) ratio calculated from D:A ratio (Fig. 5a). The histogram in Fig. 5a illustrated that all three molecules were prone to accumulating on the surface. With fluorination, surface enrichment was enhanced from 1.69 for **BTID-0F** to 1.79 for **BTID-1F** and 1.81 for **BTID-2F**, as calculated from S:C ratios. The ‘surface enrichment degree' calculated from F:C ratios were markedly higher (2.1 for **BTID-1F** and 2.6 for **BTID-2F)** than those of S:C ratios, indicating that the fluorine-substituted groups are more prone to be enriched at the active layer surface.

Subsequently, in-depth XPS measurements in the vertical direction of **BTID-0F** and **BTID-2F** blends on ZnO/ITO substrates were carried out to characterize the vertical phase distribution (Fig. 5b). We defined the active layer/ZnO interface by the appearance of a large amount of Zn element. The corrosion time of active layer was slightly different because of the material and film thickness differences. **BTID-0F** exhibited enrichments both on top surface (air/active layer) and bottom interface (active layer/ZnO). As for **BTID-2F**, surface enrichment degree was increased evidently, whereas enrichment at the bottom and bulk was suppressed. As calculated from F:C ratios, the top surface was nearly 100% of **BTID-2F** (Supplementary Methods), indicating an electron blocking layer was formed at the active layer/MoO_*x*_ interface. The more directional vertical phase distribution and formation of electron blocking layer facilitated the charge extraction/collection and recombination suppression, leading to a higher FF and *J*_sc_ in inverted devices than those of conventional devices for **BTID-2F** (Supplementary Fig. 6, Supplementary Table 4). Moreover, the vertical distribution and surface enrichment reduced recombination and increased the build-in potential of inverted devices in comparison of its conventional devices, which thereby further reduced *V*_oc_ loss. The influence of surface enrichment and vertical phase separation on the device performance was further certified in the Supplementary evidence of the role of surface enrichment and vertical phase separation (Supplementary Discussions, Supplementary Fig. 7, Supplementary Tables 3 and 5).

The surface enrichment and vertical phase distribution were further manifested by results of charge carrier mobility (Fig. 5c). All the carrier mobility were used average values measured by space-charge limited current method (Supplementary methods), obtained from thickness between 100 and 130 nm (Supplementary Fig. 8). The average hole mobility of pristine film for **BTID-0F**, **BTID-1F** and **BTID-2F** were 8.7 × 10^−4^ cm^2^ V^−1^ s^−1^, 6.4 × 10^−4^ cm^2^ V^−1^ s^−1^ and 3 × 10^−4^ cm^2^ V^−1^ s^−1^, the decrease of hole mobility with fluorination should be mainly resulted from their decreased face-on packing ratios (Supplementary Table. 3) and longer alkyl chain lengths. However, after blending of PC_71_BM, **BTID-2F** showed markedly higher hole mobility than **BTID-0F**, the average of which were 1.4 × 10^−3^ cm^2^ V^−1^ s^−1^ and 4.7 × 10^−4^ cm^2^ V^−1^ s^−1^. On the other hand, the electron mobility of **BTID-2F**/PC_71_BM blends was considerably lower than that of **BTID-0F**/PC_71_BM blends. The hole and electron mobility of **BTID-1F**/PC_71_BM were in between. The different trends for hole and electron mobility could be explained by the largest surface enrichment of **BTID-2F**, which facilitated hole transport while blocked electron transport. The increase in hole mobility of blends compared with pristine films for fluorine-substituted molecules could be explained by their increased ratios of face-on to edge-on after PC_71_BM blending (Supplementary Table 3) and favourable backbone orientation for vertical charge transport^10^.

## Discussion

Based on hierarchical morphology at lateral direction and vertical phase distribution, the morphology of active layer was schematically shown as Fig. 5d for **BTID-2F**/PC_71_BM. In lateral direction, **BTID-2F** formed a hierarchical morphology with high domain purity, composing domains with diameters of *ca.* 53.0 nm and an interpenetrating whisker network with diameters of *ca*. 12.8 nm. A similar hierarchical morphology for **BTID-1F**/PC_71_BM was observed but with a lower domain purity, while no obvious difference for two domain sizes in **BTID-0F**/PC_71_BM. In vertical direction, **BTID-2F** formed hole-transporting layers on the top interface and more directional vertical phase distributions of the active layer than those of **BTID-0F**. Therefore, fluorination-enabled optimal hierarchical morphology and surface enrichment, which could increase *V*_oc_, *J*_sc_ and FF simultaneously and thereby obtained a high PCE in inverted devices. To further verify the influence of hierarchical morphology on device performance (Supplementary Discussions), the substrate temperature was increased from 28 °C (normal) to 40 °C (hot) during film formation. Due to a faster solvent evaporation on hot substrate, the hierarchical morphology was not formed as proved by TEM images and RSoXS images (Supplementary Fig. 9). As a consequence, the device performance based on the hot substrate was decreased (Supplementary Table 6). Hence, the hierarchical morphology is one of the most important factors to obtain high performance devices.

For the molecular design, acceptor–donor–acceptor have been widely used for organic photovoltaic. To obtain a low HOMO level and a high hole mobility simultaneously, a novel π-bridge between donor and acceptor unit, 2-(thiophen-2-yl) thieno[3,2-*b*]thiophene was introduced, which presented an inner gradient-decreased electron density distribution, and facilitated the backbone charge transfer. Different from that of polymers, the end acceptor played an important role to tune molecular packing and miscibility with PC_71_BM. Therefore, fluorinated end-capped acceptor were introduced in the molecular design, which lowered surface tension and their miscibility with PCBM, As a result, the lateral and vertical morphology of the active layer was optimized. **BTID-2F** formed a hierarchical morphology in the active layer, which will inspire more investigations on the effects of π-bridges and end acceptors for high performance OSCs.

In summary, by combining traditional molecular design strategy with fine tuning surface tension and miscibility with PC_71_BM by fluorination, we designed and synthesized three novel molecules, **BTID-0F**, **BTID-1F** and **BTID-2F**, with incremental fluorine atoms. The three molecules exhibited excellent molecular properties, such as low HOMO levels, good crystallinity and high hole mobility, because of well-designed molecular structures, including new gradient-decreased electron density π-bridges and proper polarity of aromatic acceptors. Through device measurement and morphology characterization, we emphasized the importance of fluorination to hierarchical morphology, surface enrichment and directional vertical phase distribution. The optimal morphology was beneficial to charge transfer, charge collection and recombination suppression, which reduced the loss of *V*_oc_, *J*_sc_ and FF simultaneously. As a result, a record PCE of 11.3% was obtained in inverted OSCs based on small molecules, with *V*_oc_ of 0.95 V, *J*_sc_ of 15.7 mA cm^−2^ and FF of 76%.

## Methods

### Solar cell fabrication and measurements

Inverted devices were fabricated with a structure of glass/ITO/ZnO/donor:acceptor/MoO_*x*_/Ag. The ZnO precursor solution was prepared by dissolving 0.14 g of zinc acetate dihydrate (Zn(CH_3_COO)_2_·2H_2_O, 99.9%, Aldrich) and 0.5 g of ethanolamine (NH_2_CH_2_CH_2_OH, 99.5%, Aldrich) in 5 ml of 2-methoxyethanol (CH_3_OCH_2_CH_2_OH, 99.8%, J&K Scientific). Patterned ITO glass with a sheet resistance of 15 Ω sq^–1^ was purchased from CSG HOLDING Co., Ltd. The ITO-coated glass substrates were cleaned by ultrasonic treatment in detergent, DI water, acetone and isopropyl alcohol under ultrasonication for 20 min at each step. A thin layer of ZnO precursor was spin-coated at 5,000 r.p.m. onto the ITO surface. After being baked at 200 °C for 30 min, the substrates were transferred into a nitrogen-filled glove box. The mixture of small molecules and PC_71_BM with total concentration *ca*. 18 mg ml^−1^ stirred at 60 °C in chloroform for *ca.* 0.5 h until they are intensively dissolved. Subsequently, the active layer was spin-coated from chloroform solutions of blends. Finally, a layer of *ca.* 5 nm MoO_*x*_ and then an Ag layer of *ca.* 160 nm was evaporated subsequently under high vacuum (<1 × 10^−4^ Pa).

Conventional devices were fabricated with a structure of glass/ITO/PEDOT:PSS/Ca/Al. The ITO-coated glass substrates were cleaned by the same procedure with inverted devices. A thin layer of PEDOT:PSS was spin-coated at 4,000 r.p.m. onto the ITO surface. After being baked at 150 °C for 15 min, the substrates were transferred into a nitrogen-filled glove box. The mixture of small molecules and PC_71_BM with total concentration *ca*. 18 mg ml^−1^ stirred at 60 °C in chloroform for *ca.* 0.5 h until they intensively dissolved. Subsequently, the active layer was spin-coated from blend chloroform solutions of small molecules and PC_71_BM. Finally, a layer of ∼20 nm Ca and then 100 nm Al layer was evaporated under high vacuum (<1 × 10^−4^ Pa).

Device *J*–*V* characteristics was measured under AM 1.5 G (100 mW cm^−2^) using a Newport Thermal Oriel 91159A solar simulator. Light intensity is calibrated with a Newport Oriel PN 91150V Si-based solar cell. *J*–*V* characteristics were recorded using a Keithley 2400 source-measure unit. Typical cells have device areas of approximately 4 mm^2^. A mask with well-defined area was used to measure the *J*–*V* characteristics as well. EQEs were performed in air with an Oriel Newport system (Model 66902) equipped with a standard Si diode. Monochromatic light was generated from a Newport 300 W lamp source. We have used mask for BTID-2F, the errors are in 5%.

Supplementary Methods including: characterization methods: (1) molecular structure characterization and calculation (nuclear magnetic resonance, mass spectrometry spectra, discrete Fourier transform); (2) molecular properties characterization (dielectric constant, UPS, ultraviolet–vis spectra, CV, UPS, contact angle); (3) TEM, AFM, XPS and in-depth XPS characterization; (4) GIWAXS characterization; (5) RSoXs characterization; and (6) *J*_ph_ and mobility measurements. Calculation methods: (1) calculations of ionization potential of donor (IP_D_) from ultraviolet photoelectron spectroscopy (UPS); (2) calculation of the coherence length (*L*_c_) of PC_71_BM and small molecules; (3) calculation of domain size and purity from RSoXs ; (4) calculation of surface enrichment degrees; (5) calculation of surface *D:A* ratio; and (6) calculations of mobility measured from space-charge limited current. Synthesis methods including: materials and synthesis.

### Data availability

All relevant data are available from the authors.

## Additional information

**How to cite this article:** Deng, D. *et al*. Fluorination-enabled optimal morphology leads to over 11% efficiency for inverted small-molecule organic solar cells. *Nat. Commun.* **7,** 13740 doi: 10.1038/ncomms13740 (2016).

**Publisher's note:** Springer Nature remains neutral with regard to jurisdictional claims in published maps and institutional affiliations.

## Supplementary Material

Supplementary Information Supplementary Figures, Supplementary Tables, Supplementary Discussion, Supplementary Methods, Supplementary References.

Peer Review File

## Figures and Tables

**Table 1:** Optimized photovoltaic performance of inverted device based on the three molecules (device structure: ITO/ZnO/active layer/MoO_*x*_/Ag).

Donors	*V*_oc_ (V)	*J*_sc_ (mA cm^−2^)	FF (%)	PCE (%)	
				Best	Average
BTID-0F	0.93	14.0	64.0	8.30	8.21
BTID-1F	0.94	15.3	72.0	10.4	10.37
BTID-2F	0.95	15.7	76.0	11.3	11.08
