# Supplementary material for: Fluorination-enabled optimal morphology leads to over 11% efficiency for inverted small-molecule organic solar cells
Source: Nat Commun. 2016 Dec 19;7:13740. doi: 10.1038/ncomms13740 (PMC5187412; doi:10.1038/ncomms13740)
Supplement: Supplementary Information — Supplementary Figures, Supplementary Tables, Supplementary Discussion, Supplementary Methods, Supplementary References. [file ncomms13740-s1.pdf]

Supplementary Figures

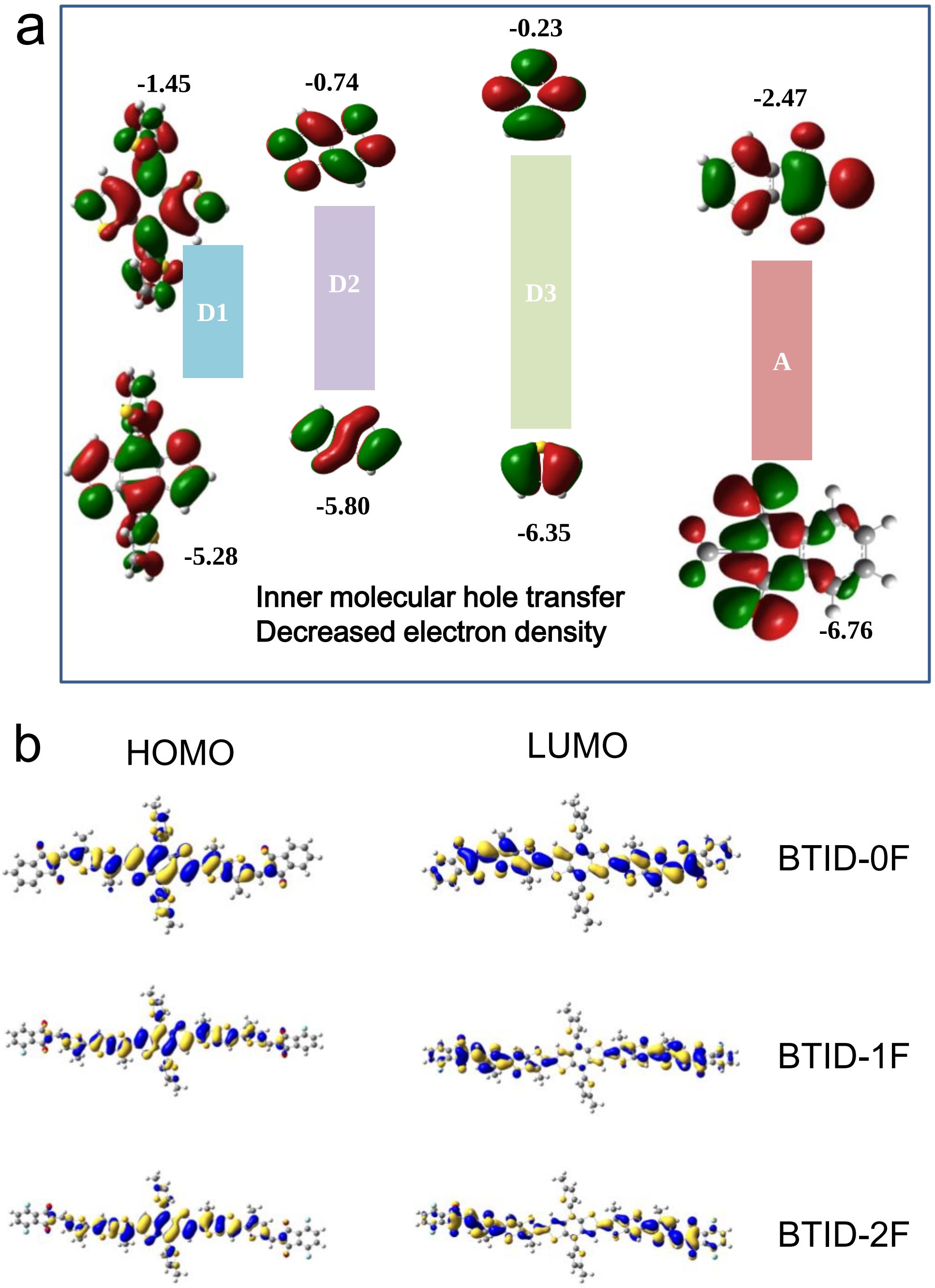

Supplementary Figure 1. Calculation results. a, HOMO and LUMO energy levels of the donor unit π-bridge and acceptor units; **b,** electron distributions of HOMO and the lowest unoccupied molecular orbital (LUMO) energy levels of **BTID-0F**, **BTID-1F** and **BTID-2F**.

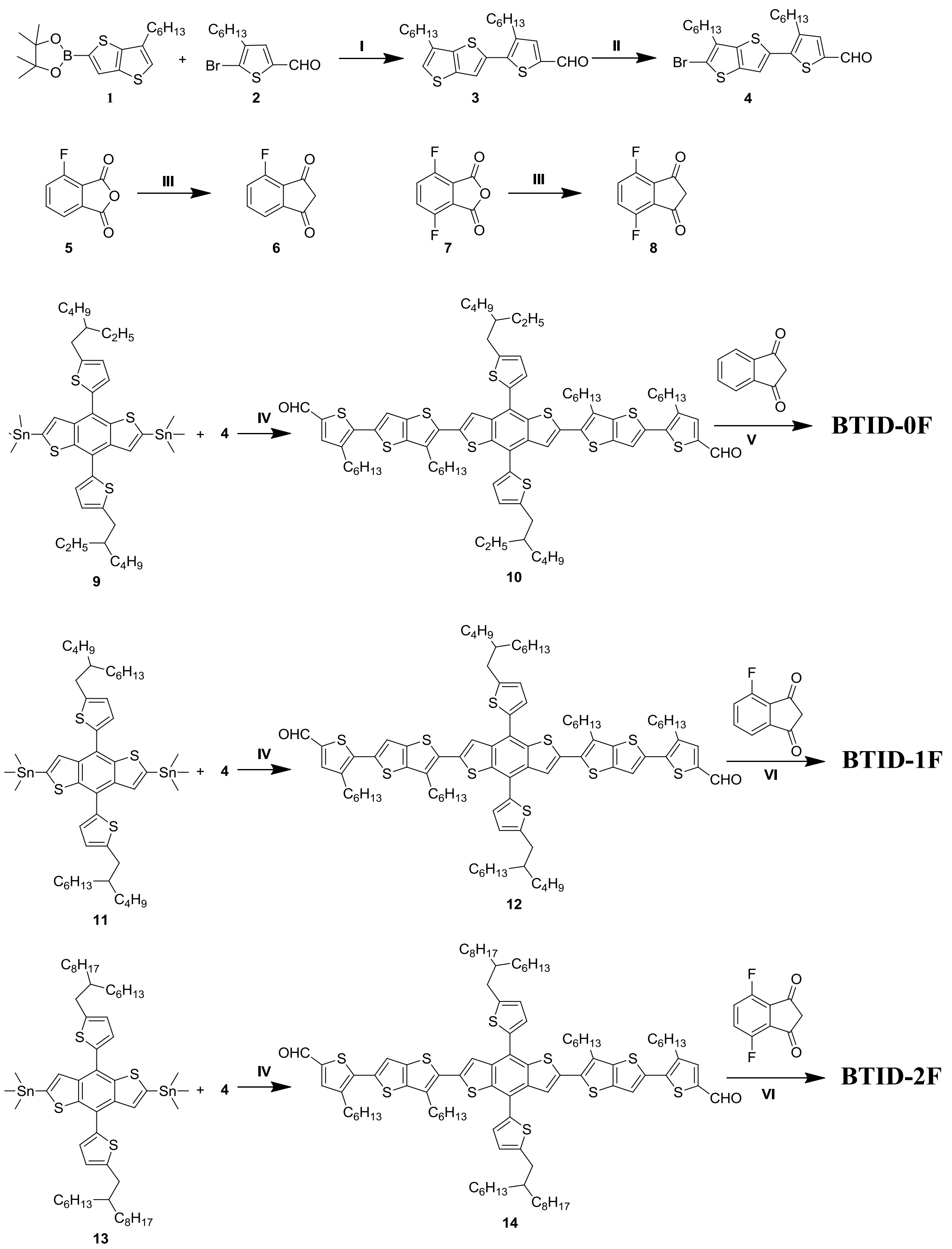

Supplementary Figure 2. Synthesis routes of the three small molecules. I. THF, H_2O, toluene, $NaHCO_3$, 85 °C, $Pd(PPh_3)_4$; II. NBS, $CHCl_3$: CH_3COOH (1:1); III. TEA, Ac_2O, *tert*-butyl acetoacetate; IV. toluene, $Pd(PPh_3)_4$; V. piperidine, $CHCl_3$; VI. TEA, $CHCl_3$.

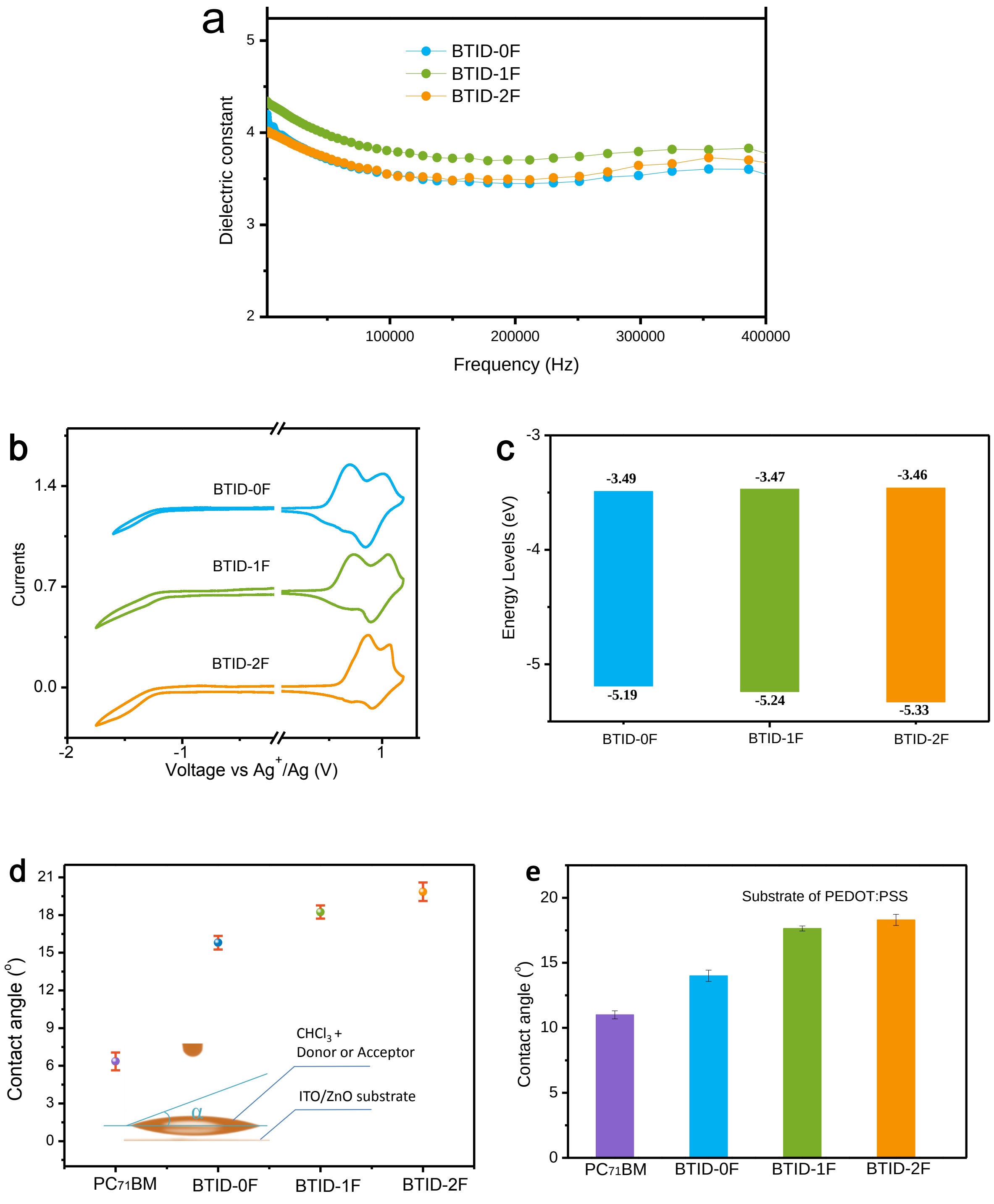

Supplementary Figure 3. Materials properties. a, dielectric constant function of frequency for the three molecules; **b,** cyclic voltammogram (CV) plots of BTID-0F, BTID-1F and BTID-2F in films; **c,** HOMO and LUMO levels calculated from CV; **d,** contact angle of small molecules on ZnO/ITO substrate, the error bar represents the measurement errors; **e,** contact angle of small molecules on PEDOT:PSS/ITO substrate, the error bar represents the measurements errors.

a

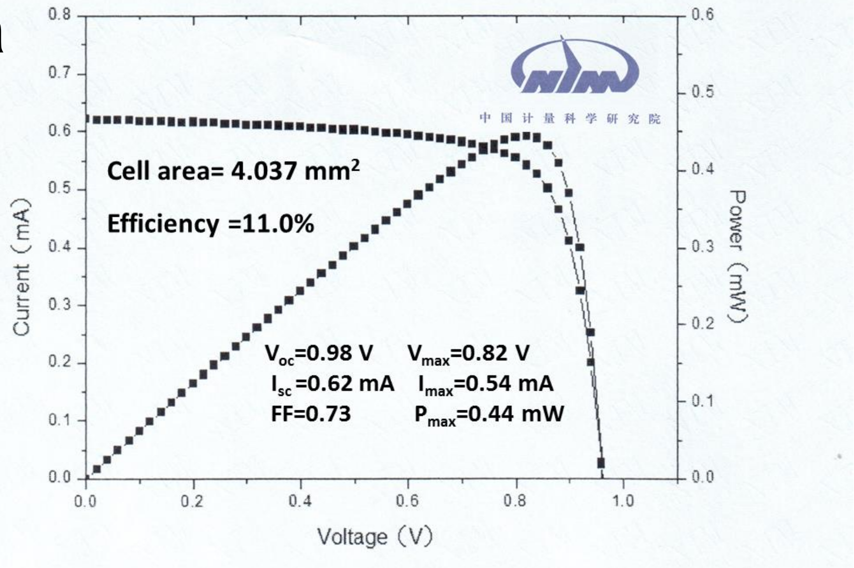

中 国 计 量 科 学 研 究 院

b

校 准 证 书
Calibration Certificate

证书编号 GXtc2016-0896
Certificate No.

客户名称 Client	国家纳米中心<br>National Center for Nanoscience and Technology (NCNST)
器具名称 Instrument	有机小分子太阳电池<br>(Small Molecular Solar Cells)
型号/规格 Type/Model	/
出厂编号 Serial No.	18-1
生产厂家 Manufacturer	/
客户地址 Address	北京市海淀区中关村北一条 11 号<br>No.11 ZhongGuanCun BeiYiTiao, 100190 Beijing, P.R. China
校准日期 Date of calibration	2016-06-01

批准人: [signature]
Approved by

中国计量科学研究院 2 校准专用章

地址: 中国 北京 北三环东路 18 号
Address: No.18 Bei San Huan Dong Lu,Beijing,P.R.China
邮编: 100029
Post Code
电话: +86-10-64525569/74
Tel
传真: +86-10-64271948
Fax
网址: http://www.nim.ac.cn
Website
电子邮箱: kehufuwu@nim.ac.cn
Email

C

中国计量科学研究院

证书编号 GXtc2016-0896
Certificate No.

中国计量科学研究院是国家最高的计量科学研究中心和国家级法定计量技术机构。1999 年授权签署了国际计量委员会（CIPM）《国家计量基(标)准和国家计量院签发的校准与测量证书互认协议》（CIPM MRA）。The National Institute of Metrology (NIM) is China's national metrology institute (NMI) and a state-level legal metrology institute. NIM is China's signatory to the Mutual Recognition of National Measurement Standards and of Calibration and Measurement Certificates Issued by National Metrology Institutes (CIPM MRA) which is arranged by the International Committee of Weights and Measures (CIPM).

中国计量科学研究院的质量管理体系符合 ISO/IEC17025 标准，通过中国合格评定国家认可委员会和亚太计量规划组织（APMP）联合评审的校准和测量能力（CMCs）在国际计量局（BIPM）关键比对数据库中公布。NIM's quality management system meets requirements of the ISO/IEC 17025. Its Calibration and Measurement Capabilities (CMCs) that are peer reviewed both by China National Accreditation Service for Conformity Assessment (CNAS) and the Asia Pacific Metrology Programme (APMP) are published in the International Bureau of Weights and Measures (BIPM) Key Comparison Database (KCDB).

2011 年，中国计量科学研究院和中国合格评定国家认可委员会就认可领域的技术评价活动签署了谅解备忘录，承认中国计量科学研究院的计量支撑作用和出具的校准/检测结果的溯源效力。NIM and CNAS signed a Memorandum of Understanding (MOU) for Recognition of Technical Assessment in Laboratory Accreditation Field in 2011, in which CNAS recognizing the technical supporting role of NIM in laboratory accreditation and the traceability of NIM's calibration / test results.

校准结果不确定度的评估和表述均符合 JJF1059 系列标准的要求。The evaluation and expression of uncertainty of the calibration results are in line with the requirements of JJF1059 series standards.

校准所依据的技术文件（代号、名称）Reference documents (Code,Name)
Measurement of photovoltaic current-voltage characteristics（IEC60904-1）
太阳电池校准规范：光电性能（NIM-ZY-GX-TT-402）(Calibration Specification of Solar Cells: Photoelectric Properties)

校准环境条件及地点 Calibration place and environment

温　度 Temperature：25　℃　　地　点 Location：　光学楼 110 室
湿　度 Humidity：45　% RH　　其　它 Others：　/

校准使用的计量基（标）准装置(含标准物质)/主要仪器
Reference Standards（Including the Reference Material）/ Instruments used

名　称<br>Name	测量范围<br>Measurement Range	不确定度/<br>准确度等级<br>Uncertainty/Accuracy	证书编号<br>Certificate No.	证书有效期至<br>Due Date<br>(YYYY-MM-DD)
太阳电池光电性能校准装置<br>Measurement Standard	I_{sc}:（0.1-10）A<br>V_{oc}: (0.1-200)V<br>P_m:(0.01-500) W	I_{sc}: 1.5%（k=2）<br>V_{oc}: 0.5% (k=2)<br>P_m:1.6% (k=2)	[2015]国量标计证字第 286 号	2019-07-05
KG5-标准太阳电池<br>KG5-Reference	I_{sc}:（0-200）mA	1.2% (k=2)	GXtc2016-0080	2017-02-26

d

中国计量科学研究院

证书编号 GXtc2016-0896
Certificate No.

校准结果
Calibration Results

1. 校准条件 Calibration Conditions:

标准太阳电池：KG5-单晶硅；

Reference Solar Cell: KG5 filterd mono-Si；

标准太阳电池的标定值：38.9 mA；

CV of Reference Solar Cell：38.9 mA；

太阳模拟器等级：AAA 级；

Solar Simulator Classification：AAA 级；

温度传感器/控制系统：无。

Teperature Sensor/Control System: None。

扫描方向：正扫

Scan Direction: Forward

2. I-V 特性参数 I-V Characteristic parameters:

以上述标准太阳电池标定太阳模拟器辐照度至 1000 W/m^2，校准被测太阳电池的 I-V 特性曲线和参数如下：

By using the above reference solar cell to calibrate the solar simulator's irradiance to 1000 W/m^2, the I-V characterisitc curve and parameters as follows:

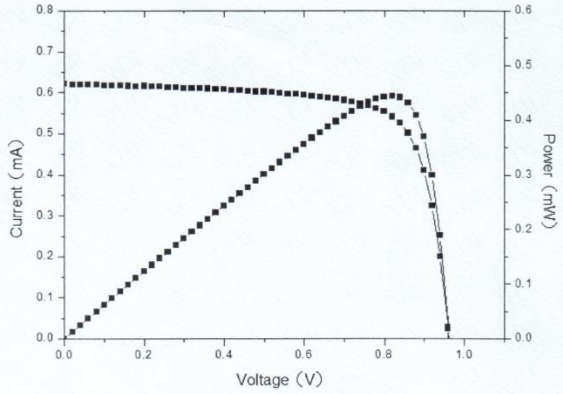

e

中 国 计 量 科 学 研 究 院

证书编号 GXtc2016-0896
Certificate No.

校 准 结 果
Calibration Results

有效面积 (mm²)	短路电流 I_{sc}(mA)	开路电压 V_{oc}(V)	最大功率 P_{max}(mW)
4.037	0.62	0.98	0.44

最大功率电流 I_{max}(mA)	最大功率电压 V_{max}(V)	填充因子 FF (%)	转换效率 (PCE) η (%)
0.54	0.82	73.0	11.0

不确定度描述 Uncertainty:

I_{sc} ：1.8%（k=2）； V_{oc}：1.2%（k=2）； P_{max}：2.2%（k=2）。

注 Note:

1. 太阳电池的有效面积为 4.037 mm²（证书编号：CDjc2016-3696）。
The certificated cell area is 4.037mm² (Certificate No.: CDjc2016-3696).
2. 此数据仅对被测样品当时状态有效。
The data apply only at the time of the test for the sample.

建议 Suggestion:

根据客户要求和校准文件的规定，通常情况下 12 个月校准一次。
According to the client or the calibration documents, the recommended calibration cycle is 12 months.

声明 Statement:

1.我院仅对加盖“中国计量科学研究院校准专用章”的完整证书负责。
NIM is ONLY responsible for the complete certificate with the calibration stamp of NIM.
2.本证书的校准结果仅对所校准的计量器具有效。
The certificate is ONLY valid for the calibrated instrument.
3.本证书用中英文两种语言表达，准确含义以中文为准。
The certificate is reported in both English and Chinese, with the Chinese version as standard.

校 准 员： [illegible]　　　　核 验 员： [illegible]

第 4 页　共 4 页

Supplementary Figure 4. Certification for inverted solar cells based on BTID-2F. a, certified *I*-*V* curve and power-*V* curve; **b-e,** details of certification.

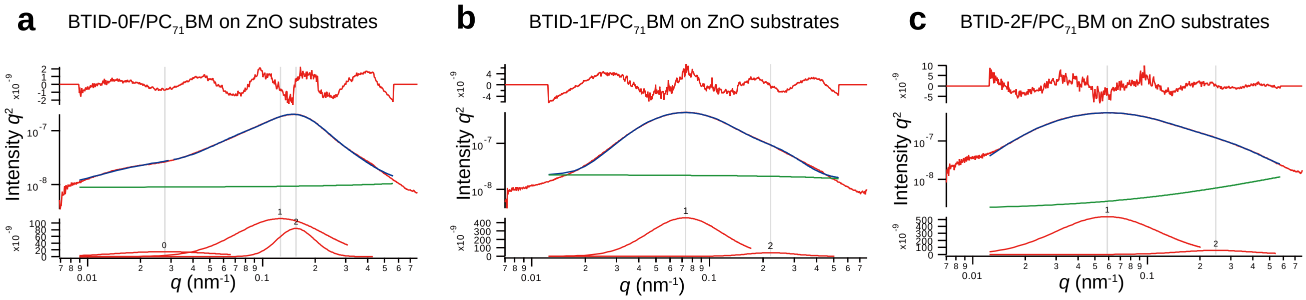

Supplementary Figure 5. Resonant soft X-ray scattering (RSoXS) fitting. a-c, fitting peaks of RSoXs on ZnO/ITO substrates.

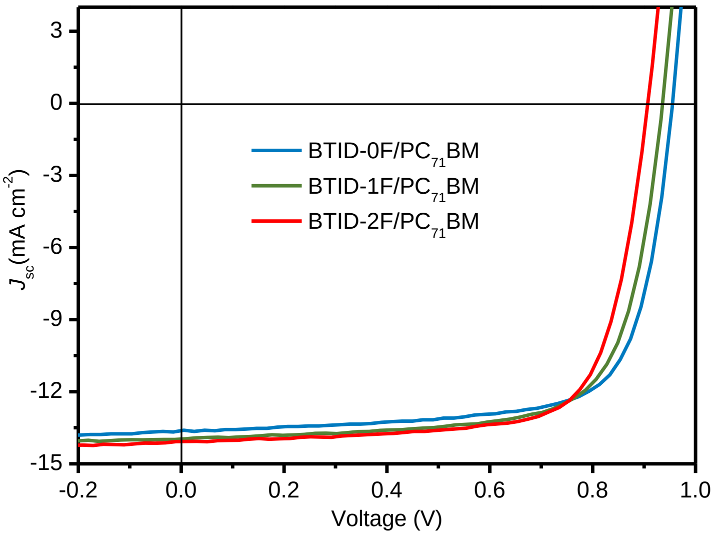

Supplementary Figure 6. *J-V* curves of conventional devices based on the three small molecules.

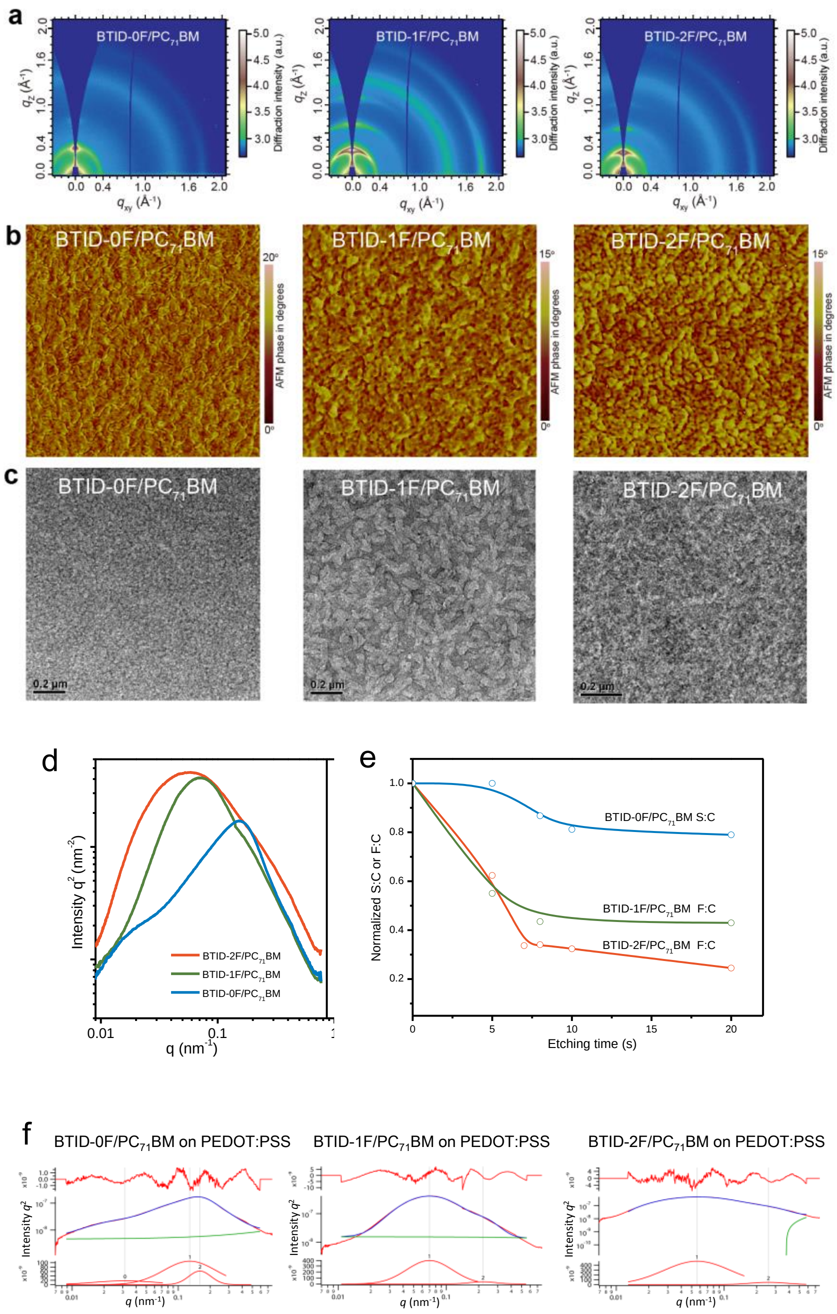

Supplementary Figure 7. Morphologies for conventional devices. a, grazing incidence wide-angle X-ray scattering (GIWAXS) images of blend films on the PEDOT: PSS/Si substrates; **b,** atomic force microscopy (AFM) phase images of blend films on the PEDOT:PSS/ITO substrate; **c,** transmission electron microscopy (TEM) images of blend films on the PEDOT:PSS/ITO substrate; **d,** R-SoXs images of blend films on the PEDOT: PSS/Si substrates; **e,** In-depth X-ray photoelectron spectroscopy (XPS) 0–20s for blend films on PEDOT:PSS/ITO substrates; **f,** fitting peaks of RSoXs on PEDOT:PSS/ITO substrates, the profile is obtained from the division value between the value of S:C after etching and the value of S:C at the film surface.

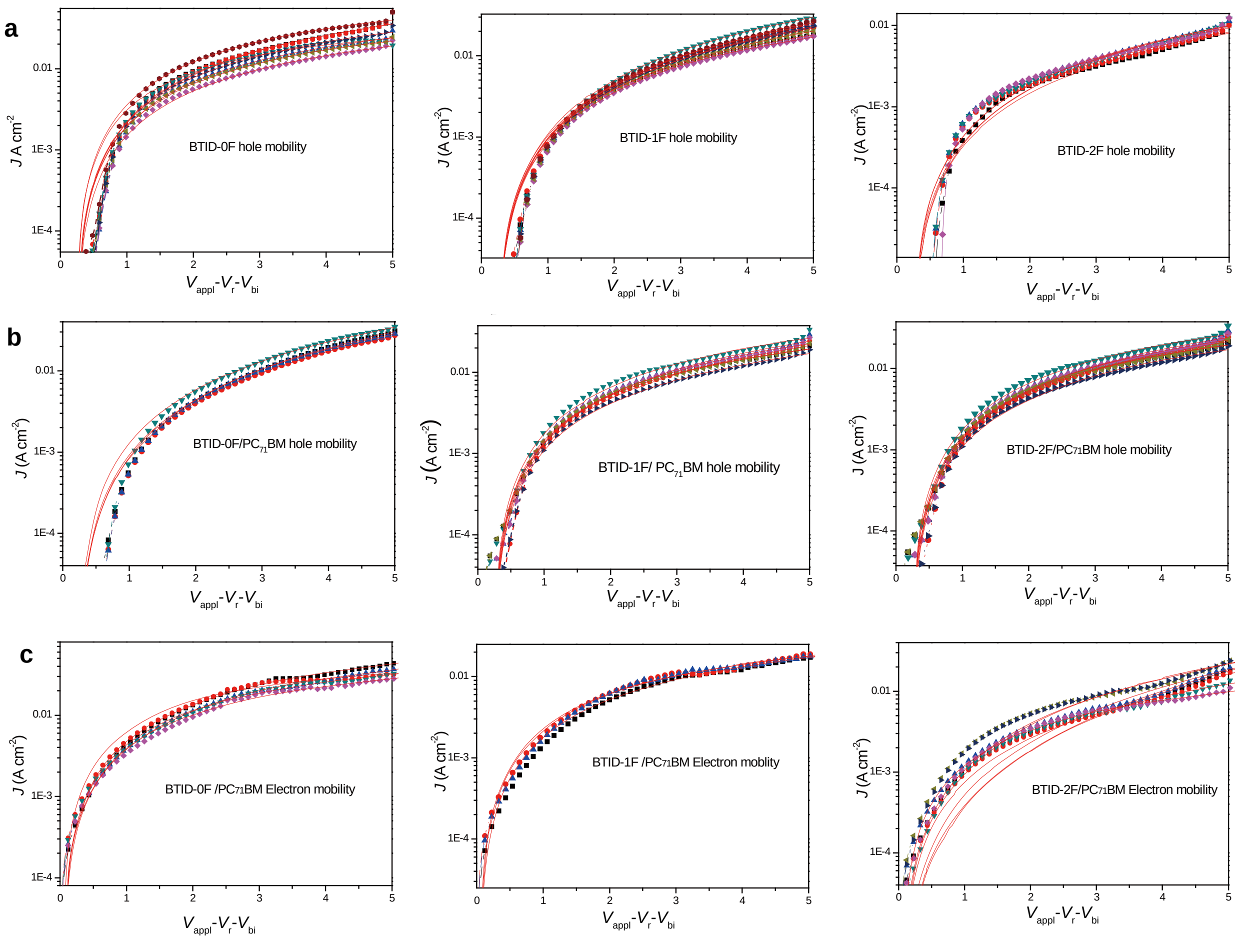

Supplementary Figure 8. Hole/electron mobility plots from SCLC methods. a, hole mobility for pristine small molecules, with thickness between 100-130 nm; **b,** hole mobility for small molecules/$PC_{71}BM$ blends, with thickness between 100-130 nm; **c,** electron mobility for small molecules/$PC_{71}BM$ blends, with thickness between 100-130 nm. All the colored scattered points are experimental data, and all the red lines are their corresponding fittting lines from mobility calculation formula (Supplementary Equation 7).

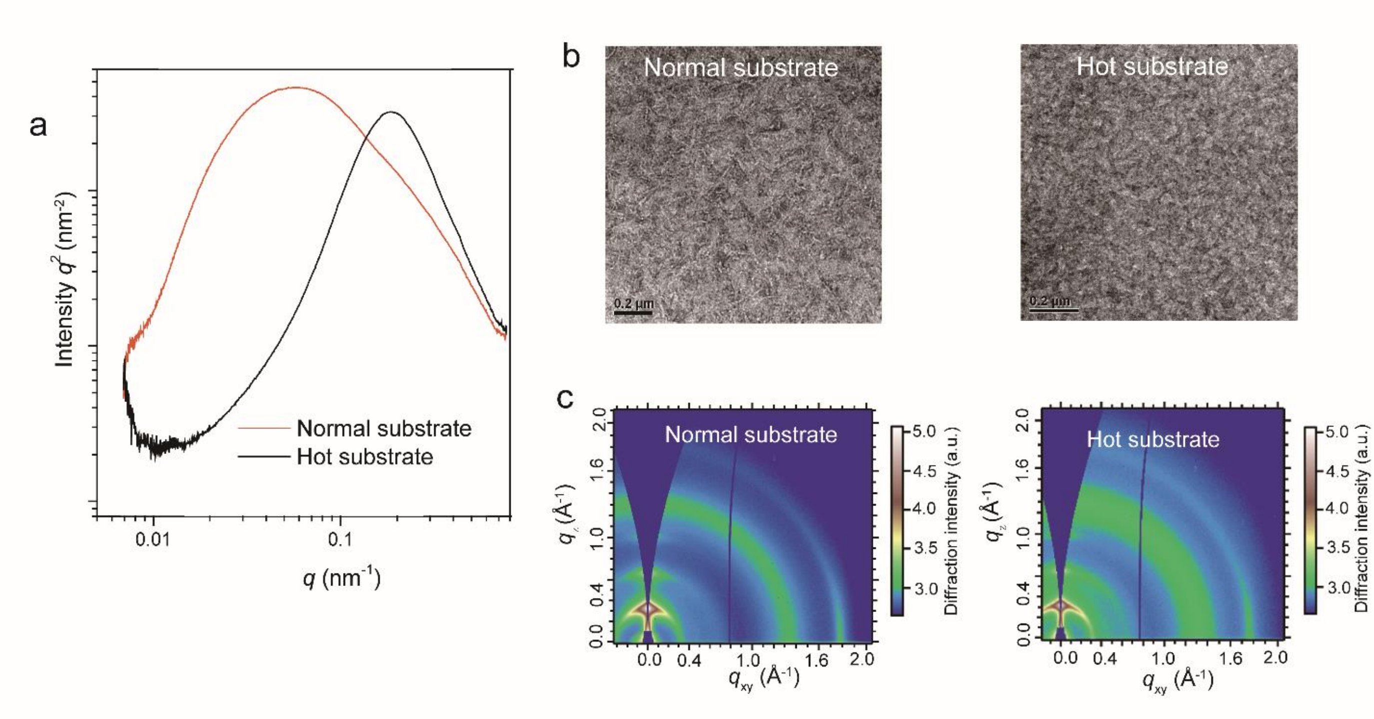

Supplementary Figure 9. Comparisons of morphologies for BTID-2F/$PC_{71}BM$ blends based on normal and hot substrates. a, comparison of R-SoXs images on normal/ hot substrates; **b,** comparison of TEM images on normal (28 ^{o}C)/hot (45 ^{o}C) substrates; **c,** comparison of GIWAXS images on normal/hot substrates.

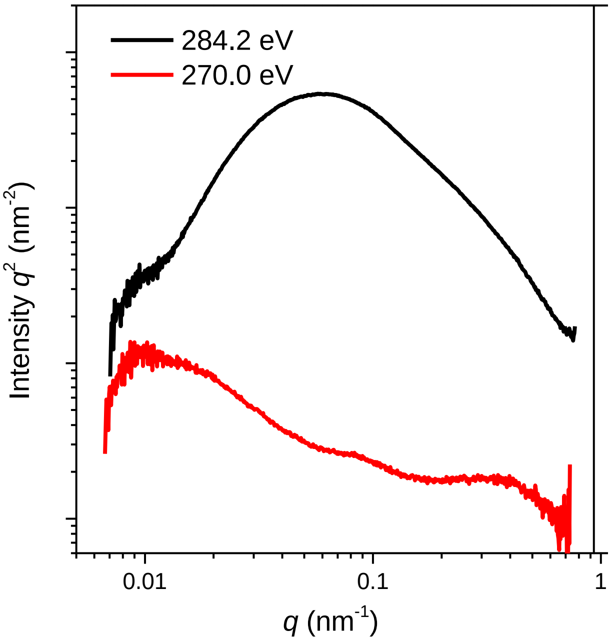

Supplementary Figure 10. RSoXs Methods. Comparison of total scattering intensity (*TSI*) based on photon energy of 270 eV and 284.2 eV choose for RSoXs.

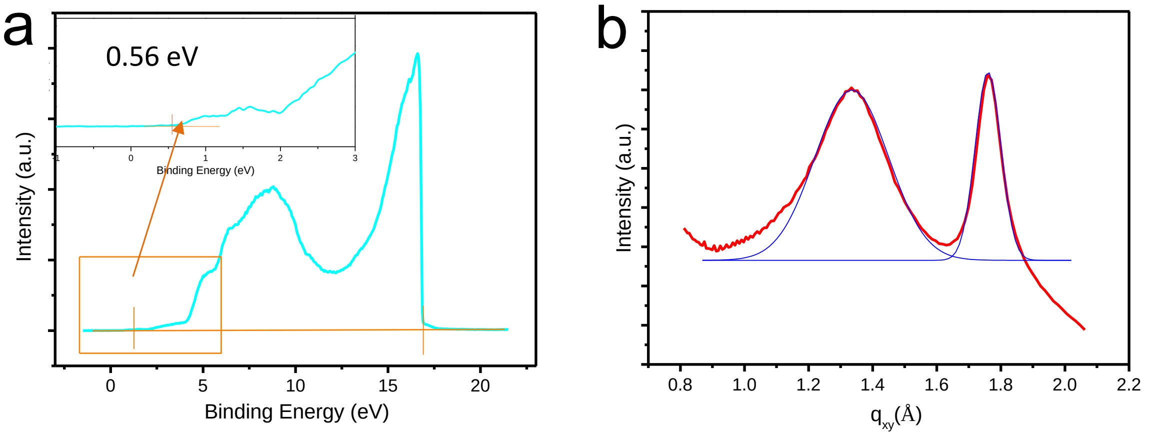

Supplementary Figure 11. a, an example of the calculation of the highest occupied molecular orbital (HOMO) energy levels from UPS; **b,** an example full width at half-maximum (FWHM) fitting of the in-plane GIWAXS peaks.

Supplementary Tables

Supplementary Table 1. Optical band-gap and dielectric constant for the three molecules.

Molecules	BTID-0F	BTID-1F	BTID-2F
E_g^{opt}	1.71 eV	1.70 eV	1.68 eV
ε_r	3.82	4.39	4.02

Supplementary Table 2. Device optimization of D:A ratios for BTID-0F/$PC_{71}BM$, BTID-1F/$PC_{71}BM$ and BTID-2F/$PC_{71}BM$.

Donors	D:A	V_{oc} (V)	J_{sc} (mA cm^{-2})	*FF* (%)	*PCE* (%)
BTID-2F	1.1:1	0.94	15.8	74.0	11.0
	1.3:1	0.95	15.7	76.0	11.3
	1.5:1	0.95	15.3	75.0	10.9
BTID-1F	1.3:1	0.94	15.5	68.5	10.0
	1.5:1	0.94	15.3	72.0	10.4
	1.7:1	0.93	15.1	65.6	9.2

	1.3:1	0.93	14.6	51.2	6.9
BTID-0F	1.5:1	0.93	14.0	64.0	8.3
	1.7:1	0.92	14.6	60.8	8.1

Supplementary Table 3. GIWAXS and RSoXs data summarization on ZnO and PEDOT:PSS substrates.

Materials	substrates	(100) *d* spacing (Å)	(010) *d* spacing (Å)	(010) Coherence length (Å)	$PC_{71}BM$ Coherence length (Å)	Relative Purity	Face on: edge on
BTID-0F		16.89	3.64	51.03			32: 68
BTID-1F		17.65	3.56	67.36			27: 73
BTID-2F		19.15	3.55	70.41			19: 81
BTID-0F/$PC_{71}BM$	ZnO	16.62	3.63	44.26	19.80	0.74	40: 60
BTID-1F/$PC_{71}BM$	ZnO	18.02	3.57	60.35	20.61	0.93	31: 69
BTID-2F/$PC_{71}BM$	ZnO	19.31	3.55	68.81	20.79	1	33: 67
BTID-0F/$PC_{71}BM$	PEDOT:PSS	16.33	3.64	44.19	19.71	0.69	34: 56
BTID-1F/$PC_{71}BM$	PEDOT:PSS	17.95	3.58	53.37	19.98	0.85	29: 71
BTID-2F/$PC_{71}BM$	PEDOT:PSS	19.44	3.56	62.84	20.70	0.97	31: 59

Supplementary Table 4. Parameters of conventional device performance based on BTID-0F/$PC_{71}BM$, BTID-1F/$PC_{71}BM$ and BTID-2F/$PC_{71}BM$.

Donors	D:A	V_{oc} (V)	J_{sc} (mA cm^{-2})	*FF* (%)	*PCE* (%)	
					Best	average
BTID-0F	1.5:1	0.96	13.7	73.0	9.52	9.47
BTID-1F	1.5:1	0.94	13.9	72.0	9.39	9.27

BTID-2F	1.3:1	0.91	14.1	73.0	9.33	9.25

Supplementary Table 5. Domain size distribution of RSoXs fitting on PEDOT:PSS

blends	Domain size 1	Domain size 2
BTID-0F	24.4	20.0
BTID-1F	44.9	14.4
BTID-2F	55.8	12.2

Supplementary Table 6. Comparison of device performance of BTID-2F/$PC_{71}BM$ on normal and hot substrates.

	V_{oc} (V)	J_{sc} (mA cm^{-2})	*FF* (%)	*PCE* (%)
Hot substrate	0.95	15.1	71	10.2
Cool substrate	0.95	15.7	76	11.3

Supplementary Discussion

Evidence of the role of surface enrichment and vertical phase separation: Conventional photovoltaic device performance and related characterization.

After fluorination, the lateral morphologies (molecular packing, TEM, domain size, domain purity) changed in the same tendency whether on ZnO or PEDOT: PSS (Supplementary Fig. 7); while in the vertical direction, electron-blocking layer formed on the active layer and the hole transported path from the active layer to surface is only favored by inverted devices. This could explain the strange phenomenon that BTID-2F obtained a higher inverted device performance while BTID-0F obtained a higher conventional device performance. Hence, the surface enrichment and vertical phase separation played an important role to enhance the inverted device performance based on BTID-2F.

Evidence of the role of hierarchical morphology

To certify the influence of hierarchical morphology on device performance, we increased the substrate temperature from 28 ^{o}C (normal) to 40 ^{o}C (hot), because increasing the substrate

temperature was to shorten the film forming time which would probably eliminate the hierarchical morphology. To our expectation, the hierarchical morphology disappeared on the hot substrate, as shown in the TEM images and RSoXs images (Supplementary Fig. 9a). Consequently, the main domain spacing was decreased from 53 to 17 nm. Though the smaller domain spacing facilitated charge separation, however, the device performance based on the cool substrate (the hierarchical morphology) was higher than that on the hot substrate (Supplementary Table. 6). In addition, the domain purity and the molecular packing was similar (Supplementary Fig. 9b-c). Hence, the hierarchical morphology would reduce the recombination loss in our materials system.

Supplementary Methods

Characterization methods

(1) Molecular structure characterization and calculation (NMR, MS spectra, DFT).

^{1}H NMR (400 MHz) and ^{13}C NMR spectra were obtained on a Bruker DMX–400 NMR Spectrometer and using tetramethylsilane as internal standard. MS spectra (MALDI–TOF–MS) were determined on a Micromass GCT–MS spectrometer. The ground-state and the first excited-state geometries of the three molecules, the HOMO and LUMO levels of donor unit, π-bridge and acceptor units were optimized by density functional theory (DFT)/time-dependent density functional theory (TDDFT) at the B3LYP/6-31G (d,p) level. For the sake of calculation, the alkyl groups were replaced by methyl. All calculations were performed in the gas phase and with Gaussian 09 program.[1-3]

(2) Molecular properties characterization (Dielectric constant, UPS, UV-Vis spectra, CV, UPS, contact angle).

Dielectric constant was measured on an electrochemical work station (VMP3 Biologic, France), according to the literature.[4] UV-Vis spectra were obtained with a JASCO–V570 spectrophotometer. UPS was measured on ITO/PEDOT: PSS substrates using an AXIS ULTRA DLD multi-functional photoelectron spectrometer. The AFM images of the blended films on ZnO/PEDOT: PSS substrates were obtained on a Nanoscope Ia AFM (Digital Instruments) in tapping mode. Electrochemical cyclic voltammetry was conducted on an electrochemical work station (VMP3 Biologic, France), with a Pt disk coated with a molecular film, a Pt plate, and an Ag/Ag^{+} electrode as working, counter,

and reference electrodes, respectively, in a 0.1 mol L^{-1} tetrabutylammonium phosphorus hexafluoride (Bu_4NPF_6) acetonitrile solution. Contact angle (θ) in solutions of **BTID-0F**, **BTID-1F**, **BTID-2F**, and $PC_{71}BM$ are measured on PEDOT: PSS/ZnO substrate by using the pendant drop method with the XG-$CAMB_3$ standard contact angle meter. Samples were dissolved in chloroform at a concentration of 5 mg/mL, and the experiment was carried out under a nearly saturated CF atmosphere. Larger θ indicates less preferential wetting.

(3) TEM, AFM, XPS and in-depth XPS characterization

All films for characterization are prepared under identical optimized conditions of device fabrication in the absence of special emphasis. The TEM images of the blended films were prepared under the same conditions as AFM: the sample on the ITO/PEDOT:PSS substrate was transferred by floating in water, whereas the sample on substrate of ITO/ZnO was transferred by floating in 0.2 M HCl. XPS and In-depth XPS and (Omicron ESCA Probe) profiling was performed on devices without the top electrode (ITO/ZnO/polymer:$PC_{71}BM$). The samples were sputtered with an Art gun at 3,000 eV and etched from the air/blend interface with a Kα X-ray photoelectron spectrometer. Integrated areas from the C 1*s*, S 2*p*, and Zn 2*p* peaks were used for data analysis.

(4) GIWAXS characterization

GIWAXS measurements were performed at the beamline of 7.3.3 at the Advanced Light Source (ALS).[5] Samples were prepared on Si/ZnO (inverted devices) or Si/PEDOT:PSS (conventional devices) substrates. The 10 keV X-ray beam was incident at a grazing angle of 0.12°–0.15° and selected to maximize the scattering intensity from the samples. Scattered X-rays were detected by using a Dectris Pilatus 2M photon counting detector. The calculations of face on: edge on ratios are according to literature.[6]

(5) RSoXs characterization

RSoXs transmission was measured at a beamline of 11.0.1.2 at the ALS.[7] Samples for RSoXs measurements were prepared on ITO/ZnO or ITO/PEDOT:PSS substrates. The samples were prepared in a similar manner as for TEM and transferred to a 1.5 mm × 1.5 mm, 100 nm thick Si_3N_4 membrane supported by a 5 mm × 5 mm, 200 µm thick Si frame (Norcada Inc.). 2D scattering patterns were collected on an in-vacuum charge-coupled device camera (Princeton Instrument PI-MTE). The sample detector distance was calibrated from diffraction peaks of a triblock copolymer poly(isoprene-b-styrene-b-2-vinyl pyridine), which presents a known spacing of 391 Å.

The beam size at the sample is ≈100 μm by 200 μm. Photon energy was selected to be 284.2 eV because of high small molecule: fullerene contrast. The scattering profiles can be processed further to extract the domain purity of an assumed two phase system through the total scattering intensity (*TSI*)[8]:

$$TSI = \int_0^\infty I(q)q^2 \mathrm{d}q = 2\pi^2 \Delta\rho_{12}^2 \upsilon_1 \upsilon_2 V \quad \text{(Equation 1)}$$

where $\Delta\rho_{12}$ is the difference in electron density between the two phases, υ_i is the material volume fraction of each domain and V is the illuminated volume. Domain contrast affect the *TSI* and thus can be used as a measure of domain purity. In resonant soft x-rays region, ρ can be extended as:

$$\rho = \frac{f_1 - if_2}{V} = \sum_j n_j (f_{1j} + f_{2j}) = \alpha E^2 (\delta - i\beta) \quad \text{(Equation 2)}$$

where $f_1 - if_2$ are the complex elemental scattering factors, n_j are the number densities for the jth element in each component, and α is a constant factor. Thus, $\Delta\rho_{12}^2 = \alpha^2 E^4 \Delta n_{12}^2$. $\Delta\rho$ indicates the contrast between domains (not materials). If the domains are purer (materials are less mixed within domains), the higher contrast will lead to higher *TSI*. As Δn scales with purity, relative domain purity can be readily obtained from *TSI*.

A photon energy of 284.2 eV was selected to provide high contrast and avoid high absorption which can lead to beam damage and fluorescence background. The contrast of *TSI* based on 270 eV and 284.2 eV has been shown in Supplementary Fig. 10.

(6) J_{ph} and mobility measurements.

For investigating the dependence of J_{sc} and J_{ph} on light intensity, the intensity of the light was modulated with a series of two neutral density filter wheels with six filters, allowing for up to 11 steps in intensity from 96.5 mW cm^{-2} to 3.52 mW cm^{-2}. Mobility measurements of pristine films and blend films were characterized by a hole-only space-charge limited current (SCLC) method with the following diode structures: ITO/PEDOT:PSS/active layer/Au for hole (Al/active layer/Al for electrons) by taking current–voltage current in the range of 0–5 V and fitting the results to a space–charge-limited form.[9-10]

Calculation methods

(1) Calculations of ionization potential of donor (IP_{D}) from ultraviolet photoelectron spectroscopy (UPS). Taking BTID-0F pristine film as an example (**Supplementary Fig. 11a**):

E_w (work function) = 21.2−16.9 = 4.3 eV

IP_D = 0.56 + (21.2−16.9) = 4.96 eV

(2) The calculation of the coherence length (L_c) of $PC_{71}BM$ and small molecules

The coherence length (L_c) of $PC_{71}BM$ and small molecules was calculated from out-of-plane (010) by Scherrer analysis:

$$Lc = 2\pi K/\Delta q \quad \text{(Equation 3)}$$

K is shape factor (0.9 is used here), Δq is FWHM of the peak. The example of fitting of FWHM has been shown in **Supplementary Fig. 11b**.

(3) Calculation of domain size and purity from RSoXs

Domain spacing is calculated from $2\pi/q$, where the q corresponds to the mode of the log-normal distribution scattering profiles, and the domain size is half of the domain spacing. The relative purity of all domains over the length-scale sample can be extracted by integrating scattering profiles to yield *ISI*. Purer average domains indicate higher *ISI*.

$$ISI = \int I_{avg} q^2(q) dq \quad \text{(Equation 4)}$$

(4) Calculation of surface enrichment degrees

Surface enrichment degrees (*SED*) were calculated by:

$$SED = \frac{\text{S:C (mesuared from XPS)}}{\text{S:C(calculated from ideal D:A)}} \quad \text{(Equation 5)}$$

or

$$SED = \frac{\text{F:C (mesuared from XPS)}}{\text{F:C(calculated from ideal D:A)}} \quad \text{(Equation 6)}$$

A detailed example for the calculation of *SED*s:

As for BTID-2F/$PC_{71}BM$, the optimized weight ratio (D:A) = 1.3:1,

To convert the value to mol ratio:

$$\frac{1.3}{1963.8} \div \frac{1}{1031.0} = 0.683$$

(1963.8 and 1031.0 are molecular weights of BTID-2F ($C_{114}H_{134}F_4O_4S_{10}$) and $PC_{71}BM$ ($C_{82}H_{14}O_2$), respectively.)

Hence,

$$\text{the ideal S:C} = \frac{0.683 \times 10}{0.683 \times 114 + 82} = 0.0427$$

The surface S:C measured from XPS = 6.97/85.9 = 0.0814

$$\mathrm{SED} = \frac{0.0814}{0.0427} = 1.91$$

(5) Calculation of surface D:A ratio.

Taking BTID-2F/$PC_{71}BM$ on ZnO as an example from F:C and supposing that D = X, then, A = 1−X,

$$\frac{F}{C} = \frac{4X}{114X + 82(1 - X)} = \frac{3.68\%}{85.92\%}$$

(3.68% and 85.92% are the F and C atom contents, respectively.)

The result: X > 1, illustrating the full surface of BTID-2F. $PC_{71}BM$ addition may slightly change the backbone orientation of BTID-2F.

(6) Calculations of mobility measured from SCLC

Charge carrier motilities were calculated by using the SCLC model considering electric-field dependence, as described by

$$J = \frac{9}{8}\varepsilon_r \varepsilon \mu V^2 \exp\left(0.89\sqrt{\frac{V}{E_0 L}}\right) / L^3 \qquad \text{(Equation 7)}$$

where J is the current density (mA cm^{-2}), L is the film thickness of the active layer (cm), μ is the hole or electron mobility, ε_r is the relative dielectric constant of the transport medium, ε_0 is the permittivity of free space (8.85×10^{-14} F cm^{-1}), and V is the internal voltage in the device. $V = V_{appl} - V_r - V_{bi}$, where V_{appl} is the applied voltage to the device, V_r is the voltage drop owing to contact resistance and series resistance across the electrodes, and V_{bi} is the built-in voltage owing to the relative work function difference of the two electrodes.

Materials and synthesis

All reagents and chemicals were purchased from Aldrich, Alfa and used as received. Solvents and other common reagents were obtained from the Beijing Chemical Plant. Toluene, chloroform and THF were freshly distilled prior to use. Other materials were used without further purification. 2-(6-hexylthieno[3,2-b]thiophen-2-yl)-4,4,5,5-tetramethyl-1,3,2-dioxaborolane (**Compound 1**), 5-bromo-4-hexylthiophene-2-carbaldehyde (**Compound 2**), (4,8-bis(5-(2-ethylhexyl)thiophen-2-yl)benzo[1,2-b:4,5-b']dithiophene-2,6-diyl)bis(trimethylstannane) (**Compound 9**), (4,8-bis(5-(2-butyloctyl)thiophen-2-yl)benzo[1,2-b:4,5-b']dithiophene-2,6-diyl)bis(trimethylstannane) (**Compound 10**), and (4,8-bis(5-(2-hexyldecyl)thiophen-2-yl)benzo[1,2-b:4,5-b']dithiophene-2,6-diyl)bis(trimethylstannan

e) (**Compound 11**) **(supplementary Fig. 2)** were synthesized according to the literature. [11-13]

Compound 3 *4-hexyl-5-(6-hexylthieno[3,2-b]thiophen-2-yl)thiophene-2-carbaldehyde*

Under an Ar atmosphere, $Pd(PPh_3)_4$ (198 mg, 2% mmol) was added to the solution of compound **1** (3 g, 8.57 mmol), compound **2** (2.5 g, 9.12 mmol), and $NaHCO_3$ (2.16 g, 25.7 mmol) in a mixed solvent of 48 mL of THF, 16 mL of distilled water, and 16 mL of toluene. Then, Ar gas was bubbled for 20 min, and the mixture was heated to 85 °C and maintained at the temperature for 48 h. After the mixture was cooled to ambient temperature, the solvent was diluted in THF; the organic layer was washed with water for three times and then dried over $MgSO_4$. After concentration, the crude product was purified with column chromatography on silica gel with a mixture of petroleum and dichloromethane (1:1) as eluent. The product was obtained as a yellow solid (3 g, 84%). MS (EI): calcd for $C_{23}H_{30}OS_3$ $[M]^+$ 418.2, found *m/z* 419. ^{1}H NMR (400 MHz, $CDCl_3$), δ: 9.84 (s, 1H), 7.61 (s, 1H), 7.41 (s, 1H), 7.05 (s, 1H), 2.81–2.85 (t, 2H), 2.72–2.76 (t, 2H), 1.69–1.80 (m, 4H), 1.30–1.42 (m, 12H), 0.89-0.91(m, 6H). ^{13}C NMR (101 MHz, $CDCl_3$) δ 182.56, 141.63, 140.91, 140.70, 138.95, 138.89, 135.80, 135.06, 123.01, 120.18, 31.60, 30.35, 29.87, 29.31, 29.13, 29.02, 28.58, 22.60, 22.59, 14.10, 14.07.

Compound 4 *5-(5-bromo-6-hexylthieno[3,2-b]thiophen-2-yl)-4-hexylthiophene-2-carbaldehyde*

In an ice bath, NBS (1.28 g, 7.2 mmol) was added in portion to the solution of compound **3** (3 g, 7.2 mmol) in a mixed solvent of 50 mL of chloroform and 50 mL of acetic acid. After addition, the mixture was warmed to ambient temperature and left undisturbed overnight. The whole mixture was poured into 50 mL of chloroform: then, the organic layer was washed with water, saturated $NaHCO_3$, and water for three times and then dried over $MgSO_4$. After concentration, the crude product was purified with column chromatography on silica gel, with petroleum and dichloromethane (3:2) as eluent, and the product was obtained as a yellow solid (3 g, 84.3%). MS (EI): calcd for $C_{23}H_{29}BrOS_3$ $[M]^+$ 496.1, found *m/z* 497. ^{1}H NMR (400 MHz, $CDCl_3$), δ: 9.84 (s, 1H), 7.61 (s, 1H), 7.32 (s, 1H), 2.78–2.83 (t, 2H), 2.73–2.77 (t, 2H), 1.67–1.74 (m, 4H), 1.31–1.39 (m, 12H), 0.88–0.91 (m, 6H). ^{13}C NMR (101 MHz, CDCl3) δ 182.54, 140.95, 140.87, 139.43, 138.88, 137.34, 135.26, 134.27, 119.54, 111.75, 22.59, 14.09, 14.07.

Compound 6 *4-fluoro-1H-indene-1, 3(2H)-dione*

A stirred solution of 4-fluoroisobenzofuran-1,3-dione (2 g, 12 mmol) and acetic anhydride (11 mL, 120 mmol) containing TEA (8.6 mL, 24 mmol) was added with *tert*-butyl acetoacetate (2.1 mL, 13 mmol). After being stirred for 6 h at room temperature, the reaction system was poured into a flask containing ice, and 5 N HCl (25 mL) was added drop-wise. The resulting mixture was stirred for 5 min, and then the flask was placed in an oil bath at 5 °C for 5 min. The flask was then cooled to room temperature, and the reaction was extracted several times with DCM (3×50 mL). The organic layers were combined, dried (sodium sulfate), filtered, and concentrated to yield a yellow solid at 60% (1.9 g). MS (EI): calcd for $C_9H_5FO_2$ $[M]^+$ 164.1, found *m/z* 165. ^{1}H NMR (400 MHz, $CDCl_3$), δ: 8.07–8.09 (d, 1H), 7.78–7.85 (m, 2H), 7.46–7.50 (t, 1H), 3.28 (s, 2H). ^{13}C NMR (101 MHz, $CDCl_3$) δ 196.15, 196.12, 193.54, 159.30, 156.64, 145.02, 137.90, 137.82, 129.98, 129.85, 123.17, 122.97, 119.27, 119.23, 45.35.

Compound 8 *4,7-difluoro-1H-indene-1,3(2H)-dione*

The procedure was the same as that for compound **6**. The product was obtained as a yellow solid with a yield of 58%. MS (EI): calcd for $C_9H_4F_2O_2$ $[M]^+$ 182.1, found *m/z* 183. ^{1}H NMR (400 MHz, $CDCl_3$), δ: 7.46–7.49 (t, 2H), 3.3 (s, 2H). ^{13}C NMR (101 MHz, $CDCl_3$) δ 192.31, 155.14, 155.09, 152.49, 152.44, 130.20, 130.14, 130.10, 130.05, 125.44, 125.32, 125.27, 125.14, 45.67.

Compound 10 5,5'-(5,5'-(4,8-bis(5-(2-ethylhexyl)thiophen-2-yl)benzo[1,2-b:4,5-b']dithiophene-2,6-diyl)bis(6-hexylthieno[3,2-b]thiophene-5,2-diyl))bis(4-hexylthiophene-2-carbaldehyde)

Under Ar protection, $Pd(PPh_3)_4$ was added to the solution of compound **4** (453 mg, 0.5 mmol) and compound **9** (496 mg, 1 mmol) in 40 mL of toluene. Then, Ar gas was bubbled for 20 min; then, the mixture was heated to 100 °C and maintained at the temperature for 12 h. After being cooled to ambient temperature, the mixture was evaporated, and the crude product was purified using column chromatography on silica gel with petroleum and dichloromethane (2:3); the product was given as a red solid (501 mg) with a yield of 71%. MALDI–TOF MS calcd for $C_{80}H_{98}O_2S_{10}$ 1410.5 found 1410.7. ^{1}H NMR (400 MHz, $CDCl_3$), δ: 9.84 (s, 2H), 7.73 (s, 2H), 7.62 (s, 2H), 7.39 (s, 2H), 7.36–7.37 (m, 2H), 6.92–6.93 (m, 2H), 2.98–3.01 (t, 4H), 2.81–2.89 (m, 8H), 1.67–1.81 (m, 12H), 1.32–1.40 (m, 36H), and 0.88–0.98 (m, 24H). ^{13}C NMR (101 MHz, $CDCl_3$) δ 182.49, 146.08, 142.79, 141.28, 140.84, 140.72, 139.21, 138.93, 137.59, 137.25, 136.83, 136.76, 136.31, 133.77, 132.81, 127.87, 125.52, 123.53, 122.13, 119.74, 41.51, 34.32, 32.56, 31.62, 31.57, 30.31, 29.43, 29.25, 29.16,

29.02, 28.96, 25.74, 23.07, 22.64, 22.61, 14.22, 14.12, 14.08, 10.93.

Compound 12 5,5'-(5,5'-(4,8-bis(5-(2-butyloctyl)thiophen-2-yl)benzo[1,2-b:4,5-b']dithiophene-2,6-diyl)bis(6-hexylthieno[3,2-b]thiophene-5,2-diyl))bis(4-hexylthiophene-2-carbaldehyde)

The synthesized process was the same as that of compound **10**. The product was obtained as a red solid with a yield of 63%. MALDI–TOF MS calcd for $C_{88}H_{114}O_2S_{10}$ 1522.6 found 1523.4. ^{1}H NMR (400 MHz, $CDCl_3$), δ:9.85 (s, 2H), 7.74 (s, 2H), 7.62 (s, 2H), 7.43 (s, 2H), 7.36-7.39 (t, 2H), 6.91–6.92 (m, 2H), 2.98–3.02 (t, 4H), 2.83–2.98 (m, 8H), 1.69–1.81 (m, 12H), 1.29–1.43 (m, 84H), 0.90–0.98 (m, 24H). ^{13}C NMR (101 MHz, $CDCl_3$) δ 182.52, 146.10, 142.78, 141.29, 140.88, 140.77, 139.27, 138.93, 137.62, 137.26, 136.86, 136.74, 136.29, 133.74, 132.85, 127.85, 125.53, 123.58, 122.23, 119.77, 40.07, 34.73, 33.44, 33.06, 31.95, 31.61, 31.57, 30.32, 29.73, 29.40, 29.22, 29.15, 29.02, 28.93, 26.70, 23.07, 22.72, 22.64, 22.60, 14.21, 14.14, 14.12, 14.07.

Compound 14 5,5'-(5,5'-(4,8-bis(5-(2-hexyldecyl)thiophen-2-yl)benzo[1,2-b:4,5-b']dithiophene-2,6-diyl)bis(6-hexylthieno[3,2-b]thiophene-5,2-diyl))bis(4-hexylthiophene-2-carbaldehyde)

The synthesis process was the same with compound **10** with a yield of 74%. MALDI–TOF MS calcd for $C_{96}H_{130}O_2S_{10}$ 1634.7 found 1635.2. ^{1}H NMR (400 MHz, $CDCl_3$), δ: 9.85 (s, 2H), 7.74 (s, 2H), 7.62 (s, 2H), 7.41 (s, 2H), 7.36–7.39 (m, 2H), 6.91–6.92 (m, 2H), 2.98–3.02 (t, 4H), 2.83-2.91 (m, 8H), 1.69-1.81 (m, 12H), 1.24-1.42 (m, 70H), 0.90-0.96 (m, 24H). ^{13}C NMR (101 MHz, $CDCl_3$) δ 182.50, 146.08, 142.78, 141.28, 140.86, 140.77, 139.26, 138.91, 137.63, 137.26, 136.86, 136.74, 136.29, 133.74, 132.83, 127.85, 125.52, 123.57, 122.21, 119.76, 40.10, 34.72, 33.42, 31.94, 31.61, 31.57, 30.32, 30.07, 29.74, 29.70, 29.42, 29.39, 29.23, 29.15, 29.02, 26.72, 26.70, 22.72, 22.71, 22.64, 22.60, 14.13, 14.08.

BTID-0F

Under Ar protection, five drops of piperidine was added into the mixture of compound **12** (200 mg, 0.14 mmol) and 1H-indene-1,3(2H)-dione (207 mg, 1.4 mmol). After being stirred for 24 h at ambient temperature, the mixture was poured into water and extracted using $CHCl_3$. The organic layer was washed with brine and water and then dried over $MgSO_4$. After being concentrated, the crude product was purified by using column chromatography on silica gel, with a mixture of $CHCl_3$ and petroleum (2:3) as eluent, and then recrystallized with chloroform and hexane to yield the target

compound (150 mg, 45%) as a dark solid. MALDI–TOF MS calcd for $C_{98}H_{106}O_4S_{10}$ 1666.5 found 1689.0, ^{1}H NMR (400 MHz, $CHCl_3$): 7.81–7.83 (t, 4H), 7.74–7.75 (m, 4H), 7.47 (s, 2H), 7.37 (m, 2H), 7.06–7.34 (m, 6H), 6.94 (m, 2H), 2.84–2.97 (m, 12H), 1.72–1.79 (m, 12H), 1.26–1.45 (m, 38H), 0.92–1.00 (m, 24H). ^{13}C NMR (101 MHz, $CDCl_3$) δ 190.05, 187.82, 146.17, 145.43, 144.99, 143.77, 143.29, 142.35, 142.21, 141.70, 141.30, 141.15, 140.65, 139.88, 139.37, 138.98, 138.07, 137.43, 137.41, 137.00, 136.88, 135.74, 135.44, 135.24, 135.09, 134.95, 134.84, 134.73, 134.56, 134.21, 133.67, 132.97, 132.94, 132.90, 132.85, 131.41, 127.97, 125.54, 124.28, 123.63, 122.99, 122.86, 122.20, 119.79, 41.64, 34.47, 32.72, 31.67, 31.63, 30.16, 29.50, 29.43, 29.25, 29.07, 25.92, 23.09, 22.67, 22.63, 14.17, 14.09, 14.04, 10.98.

BTID-1F

Under the protection of Ar, five drops of TEA was added in the mixture of compound **10** (200 mg, 0.13 mmol) and 4-fluoro-1H-indene-1, 3(2H)-dione (215 mg, 1.3 mmol). After being stirred for 24 h at ambient temperature, the mixture was poured into water and extracted with $CHCl_3$. The organic layer was washed with brine and water and then dried over $MgSO_4$. After concentration, the crude product was purified with column chromatography on silica gel, with a mixture of $CHCl_3$ and petroleum (2:1) as eluent, and then recrystallized with chloroform and hexane to yield the target compound (150 mg, 45%) as a dark solid. MALDI–TOF MS calcd for $C_{106}H_{120}F_2O_4S_{10}$ 1814.6 found 1815.3, ^{1}H NMR (400 MHz, $CHCl_3$) δ: 7.85–7.86 (m, 4H), 7.72–7.76 (m, 6H), 7.49 (s, 2H), 7.38 (s, 4H), 6.94 (s, 2H), 2.87–2.99 (m, 12H), 1.76–1.79 (m, 12H), 1.31–1.45 (m, 54H), 0.83–0.91 (m, 24H). ^{13}C NMR (101 MHz, $CDCl_3$) δ: 189.20, 186.91, 186.65, 146.07, 143.63, 143.42, 142.39, 141.29, 139.19, 137.94, 136.85, 136.73, 134.77, 134.37, 132.79, 127.91, 125.53, 123.52, 122.10, 119.90, 118.91, 40.07, 34.74, 33.46, 33.06, 31.96, 31.64, 31.60, 30.05, 29.75, 29.49, 29.25, 28.98, 28.93, 26.70, 23.09, 22.73, 22.68, 22.63, 14.24, 14.15, 14.09.

BTID-2F

The synthesized process was the same with compound 1F. MALDI–TOF MS calcd for $C_{114}H_{134}F_4O_4S_{10}$ 1962.7 found 1963.8. ^{1}H NMR (400 MHz, CDCl3) δ: 7.85–7.86 (m, 4H), 7.72 (s, 2H), 7.49 (s, 2H), 7.35–7.39 (m, 6H), 6.94 (s, 2H), 2.86–2.96 (m, 12H), 1.72–1.79 (m, 12H), 1.26–1.45 (m, 70H), 0.83–0.91 (m, 24H). ^{13}C NMR (101 MHz, $CDCl_3$) δ: 185.89, 185.56, 147.08, 146.09, 143.60, 141.47, 141.46, 139.47, 139.24, 138.00, 137.53, 137.23, 136.87, 136.70, 136.55, 134.69, 134.66, 134.61, 134.55, 132.83, 128.10, 127.97, 127.91, 125.54, 123.59, 122.25, 122.19, 120.11,

99.98, 40.10, 34.73, 33.42, 31.95, 31.94, 31.63, 31.59, 30.07, 30.02, 29.74, 29.70, 29.49, 29.38, 29.24, 28.99, 26.71, 26.69, 22.72, 22.70, 22.68, 22.63, 14.15, 14.12, 14.08.

Supplementary References

1 Gaussian 09 D.01, F., M. J.; Trucks, G. W.; Schlegel, H. B.; Scuseria, G. E.; Robb, M. A.; Cheeseman, J. R.; Montgomery, Jr., J. A.; Vreven, T.; Kudin, K. N.; Burant, J. C.; Millam, J. M.; Iyengar, S. S.; Tomasi, J.; Barone, V.; Mennucci, B.; Cossi, M.; Scalmani, G.; Rega, N.; Petersson, G. A.; Nakatsuji, H.; Hada, M.; Ehara, M.; Toyota, K.; Fukuda, R.; Hasegawa, J.; Ishida, M.; Nakajima, T.; Honda, Y.; Kitao, O.; Nakai, H.; Klene, M.; Li, X.; Knox, J. E.; Hratchian, H. P.; Cross, J. B.; Bakken, V.; Adamo, C.; Jaramillo, J.; Gomperts, R.; Stratmann, R. E.; Yazyev, O.; Austin, A. J.; Cammi, R.; Pomelli, C.; Ochterski, J. W.; Ayala, P. Y.; Morokuma, K.; Voth, G. A.; Salvador, P.; Dannenberg, J. J.; Zakrzewski, V. G.; Dapprich, S.; Daniels, A. D.; Strain, M. C.; Farkas, O.; Malick, D. K.; Rabuck, A. D.; Raghavachari, K.; Foresman, J. B.; Ortiz, J. V.; Cui, Q.; Baboul, A. G.; Clifford, S.; Cioslowski, J.; Stefanov, B. B.; Liu, G.; Liashenko, A.; Piskorz, P.; Komaromi, I.; Martin, R. L.; Fox, D. J.; Keith, T.; Al-Laham, M. A.; Peng, C. Y.; Nanayakkara, A.; Challacombe, M.; Gill, P. M. W.; Johnson, B.; Chen, W.; Wong, M. W.; Gonzalez, C.; and Pople, J. A (2010)

2 Karolewski, A.; Stein, T.; Baer, R.; Kummel, S. Communication: Tailoring the optical gap in light-harvesting molecules. *J. Chem. Phys. 134*, 151101 (2011).

3 Stein, T.; Eisenberg, H.; Kronik, L.; Baer, R. Fundamental Gaps in Finite Systems from Eigenvalues of a Generalized Kohn-Sham Method. *Phys. Rev. Lett.* ***105***, 266802 (2010).

4 Leblebici, S. Y., Chen, T. L., Olalde-Velasco, P., Yang, W. & Ma, B. Reducing Exciton Binding Energy by Increasing Thin Film Permittivity: An Effective Approach to Enhance Exciton Separation Efficiency in Organic Solar Cells. *ACS Appl. Mater. Interfaces* **5**, 10105-10110 (2013).

5 Vohra, V., Kawashima, K., Kakara, T., Koganezawa, T., Osaka, I., Takimiya, K. & Murata, H. Efficient Inverted Polymer Solar Cells Employing Favourable Molecular Orientation. *Nature Photon.* **9**, 403-408 (2015).

6 A. Hexemer, *et al*, presented at *14th Int. Conf. Small-Angle Scattering (SAS09)*, Oxford, England, September 13–18 (2009).

7 E. Gann, A. T. Young, *et al*, Soft x-ray scattering facility at the Advanced Light Source with real-time data processing and analysis. *Rev. Sci. Instrum. 83*, 045110 (2012)

8 B. A. Collins, Z. Li, J. R. Tumbleston, E. Gann, C. R. McNeill, H. Ade, Absolute measurement of domain composition and nanoscale size distribution explains performance in PTB7: $PC_{71}BM$ solar cells. *Adv. Energy*

Mater. 3, 65 (2013).

9 G. Malliaras, J. Salem, P. Brock, C. Scott, Electrical characteristics and efficiency of single-layer organic light-emitting diodes. *Physical Review B*, 58, R13411 (1998).

10 A. B. Tamayo, B. Walker, T.-Q. Nguyen, A low band gap, solution processable oligothiophene with a diketopyrrolopyrrole core for use in organic solar cells. *J. Phys. Chem. C*. 112, 11545 (2008).

11 He, M. & Zhang, F. Synthesis and Structure of Alkyl-Substituted Fused Thiophenes Containing up to Seven Rings. *J. Org. Chem.* **72**, 442-451 (2007).

12 Huo, L., Zhang, S., Guo, X., Xu, F., Li, Y. & Hou, J. Replacing Alkoxy Groups with Alkylthienyl Groups: A Feasible Approach To Improve the Properties of Photovoltaic Polymers. *Angew. Chem. Int. Ed.* **123**, 9871-9876 (2011).

13 Gautrot, J. E., Hodge, P., Cupertino, D. & Helliwell, M. 2,6-Diaryl-9,10-anthraquinones as models for electron-accepting polymers. *New J. Chem.* **31**, 1585-1593 (2007).
